# A Stochastic Intracellular Model of Anthrax Infection With Spore Germination Heterogeneity

**DOI:** 10.3389/fimmu.2021.688257

**Published:** 2021-08-23

**Authors:** Bevelynn Williams, Martín López-García, Joseph J. Gillard, Thomas R. Laws, Grant Lythe, Jonathan Carruthers, Thomas Finnie, Carmen Molina-París

**Affiliations:** ^1^Department of Applied Mathematics, School of Mathematics, University of Leeds, Leeds, United Kingdom; ^2^CBR Division, Defence Science and Technology Laboratory, Salisbury, United Kingdom; ^3^Emergency Response Department, Public Health England, Salisbury, United Kingdom; ^4^T-6, Theoretical Biology and Biophysics, Theoretical Division, Los Alamos National Laboratory, Los Alamos, NM, United States

**Keywords:** *Bacillus anthracis*, Markov process, intracellular model, spore germination, rupture size distribution, approximate Bayesian computation, time to macrophage rupture, dose-response

## Abstract

We present a stochastic mathematical model of the intracellular infection dynamics of *Bacillus anthracis* in macrophages. Following inhalation of *B. anthracis* spores, these are ingested by alveolar phagocytes. Ingested spores then begin to germinate and divide intracellularly. This can lead to the eventual death of the host cell and the extracellular release of bacterial progeny. Some macrophages successfully eliminate the intracellular bacteria and will recover. Here, a stochastic birth-and-death process with catastrophe is proposed, which includes the mechanism of spore germination and maturation of *B. anthracis*. The resulting model is used to explore the potential for heterogeneity in the spore germination rate, with the consideration of two extreme cases for the rate distribution: continuous Gaussian and discrete Bernoulli. We make use of approximate Bayesian computation to calibrate our model using experimental measurements from *in vitro* infection of murine peritoneal macrophages with spores of the Sterne 34F2 strain of *B. anthracis*. The calibrated stochastic model allows us to compute the probability of rupture, mean time to rupture, and rupture size distribution, of a macrophage that has been infected with one spore. We also obtain the mean spore and bacterial loads over time for a population of cells, each assumed to be initially infected with a single spore. Our results support the existence of significant heterogeneity in the germination rate, with a subset of spores expected to germinate much later than the majority. Furthermore, in agreement with experimental evidence, our results suggest that most of the spores taken up by macrophages are likely to be eliminated by the host cell, but a few germinated spores may survive phagocytosis and lead to the death of the infected cell. Finally, we discuss how this stochastic modelling approach, together with dose-response data, allows us to quantify and predict individual infection risk following exposure.

## Introduction

Anthrax is an infectious disease, caused by the bacterium *Bacillus anthracis*. Under adverse conditions, *B. anthracis* forms a dormant spore, unable to replicate. These spores monitor their environment, and when favourable conditions are detected, such as the nutrient content of a host, the spores begin to germinate into vegetative bacteria, which can replicate inside the host (1). *B. anthracis* spores in the surrounding air inhaled into the lungs can lead to inhalational anthrax, which is usually fatal if not rapidly detected and treated (2). Even with treatment, fatality rates for inhalational anthrax can be rather high if the treatment is not started early enough after exposure and symptoms onset. *B. anthracis* spores can be produced and preserved, making inhalational anthrax a potential bio-terror threat (3).

Inhalational anthrax is initiated by ungerminated, dormant *B. anthracis* spores, inhaled by a host. The spores travel through the air passages and eventually reach the alveoli of the lungs. There is some evidence that spores may be able to germinate extracellularly in the lungs (4). However the generally accepted model of inhalational anthrax infection is the Trojan horse model, which assumes that ungerminated spores must be engulfed by alveolar phagocytes before they begin to germinate (5). Once the spores have been phagocytosed, the infected phagocytes migrate into the nearby lymph nodes in the mediastinum. Macrophages play a key role in the early infection stages of anthrax, since they can induce microbicidal defences against intracellular pathogens and help to clear the infection (6). It has also been shown that 1-2 hours after phagocytosis, newly germinated bacteria are able to escape from macrophage phagosomes and begin to replicate in the cytosol, before being released from the macrophage into the extracellular environment when the host cell ruptures and dies (7). Dendritic cells are also thought to play a role in the trafficking of *B. anthracis* to the lymph nodes during the early stages of infection, since they have been found to readily engulf *B. anthracis* spores (8, 9), and transport them to the lymph nodes in a mouse model of inhalational infection (10). Once an infected host cell ruptures, the extracellular bacteria continue to multiply, leading to oedema and haemorrhage of the mediastinal lymph nodes, and large amounts of fluid in the pleural cavity, which can severely affect breathing (11). The bacteria can also spread into the bloodstream and other organs to establish a systemic infection (12). One of the characteristic virulence factors of *B. anthracis* is the production of toxins. The two anthrax toxins, oedema toxin and lethal toxin, cause different cellular responses and are essential factors for the survival of bacteria in the infected host. Lethal toxin disrupts cell signalling pathways of macrophages and some other cells, leading to cell death, whereas oedema toxin inhibits the phagocytosis of bacteria by neutrophils (13). In some cell types, oedema toxin also increases the levels of cyclic adenosine monophosphate, which is a chemical messenger that plays a major role in controlling many intracellular processes. Together, the anthrax toxins cause suppression of the host’s immune system, often leading to death of the host. Another important factor for the survival of *B. anthracis* bacteria in the host is the antiphagocytic capsule, which allows extracellular, vegetative bacteria to avoid eradication by the immune system by preventing the bacteria being phagocytosed and destroyed (14).

In this paper, we propose a stochastic model for the intracellular infection dynamics of inhalational anthrax, which adapts and extends the deterministic one of Pantha et al. (15). The model by Pantha et al. is a system of ordinary differential equations (ODEs) representing the interaction between macrophages and *B. anthracis* spores, and considers two intracellular bacterial populations: newly germinated bacteria, which are susceptible to macrophage killing but unable to replicate, and vegetative bacteria, which are susceptible to macrophage killing and capable of replicating. Spores germinate into newly germinated bacteria, and the newly germinated bacteria must mature into vegetative bacteria before they can begin to replicate. Similarly to the model in (15), our model considers the germination of spores, replication of bacteria, and killing of bacteria by the host cell. Still, we make use of a stochastic approach, instead of a deterministic one, to describe the population dynamics of spores and bacteria. We follow the methods recently developed by Carruthers et al. (16) for *Francisella tularensis* infection, extended here to include spores and spore germination, since *B. anthracis* is a spore-forming bacteria and *F. tularensis* is not.

An important addition in our stochastic model is the consideration of macrophage rupture, not explicitly considered in (15). The rupture of host cells and the release of bacteria into the extracellular environment is an important mechanism in the pathogenesis of anthrax. Thus, incorporating this event into the model allows one to better understand both the timescales of macrophage rupture, and the rupture size distribution (*i.e.*, the number of vegetative bacteria released upon rupture). These summary statistics can then play an important role when considering within-host infection dynamics, such as in the model by Day et al. (17), or when linking to dose-response data (18), as considered in the *Discussion* section. In the same way as Carruthers et al. (16), we assume that an infected macrophage’s rupture probability per unit time is proportional to its bacterial load. Thus, cells with a high bacterial load at a given time are more likely to rupture than those with a lower one. This hypothesis is supported by Ruthel et al. (19), who suggest that the intracellular bacterial load may be a contributing factor to whether a macrophage will survive an infection.

A second improvement in our model is the consideration of spore germination heterogeneity. Motivation for this comes from the work by Setlow (1, 20, 21), where it is shown that germination rates are highly heterogeneous for the *Bacillus* species spores, with germination times ranging from a few minutes to longer than 24 hours. It is thought that this spore germination heterogeneity is primarily due to variation in the germinant receptor levels between individual spores. Setlow mentions that spores with very low germinant receptor levels germinate extremely slowly and are termed superdormant (1, 20, 21). Hence, in our model we explore two hypotheses for this heterogeneity. The first hypothesis is that the germination rate is continuously distributed in a population of spores and follows a truncated normal distribution. The second hypothesis is that the population of spores can be roughly split into two discrete groups, with different germination rates, where one group corresponds to the spores with “average” germinant receptor levels, and the other corresponds to the superdormant spores.

Our resulting stochastic model is a linear birth-and-death process with catastrophe, extended to account for spore germination heterogeneity. For this model, we show how to compute the probability of either rupture or recovery of the infected cell and the conditional mean times taken to reach these fates. Furthermore, we adapt some of the results from (16) in order to compute the probability distribution of rupture times, which is shown to be proportional to the mean number of vegetative bacteria in the cell over time. We are also able to compute the probability distribution of the rupture size, which is the number of bacteria that are eventually released into the extracellular environment from one single infected cell. We carry out parameter calibration by means of Approximate Bayesian Computation Sequential Monte Carlo (ABC-SMC) (22), and by making use of the spore and bacterial counts experimentally measured by Kang et al. (23). We then present numerical results to quantify the implications of the calibrated model. Finally, we discuss the possible application of our intracellular model to explain dose-response data for anthrax infection and explore the relationship between mechanistic approaches and existing dose-response models.

## Materials and Methods

### Stochastic Model for the Dynamics of Spores and Bacteria in a Single Infected Cell

**Figure 1:**
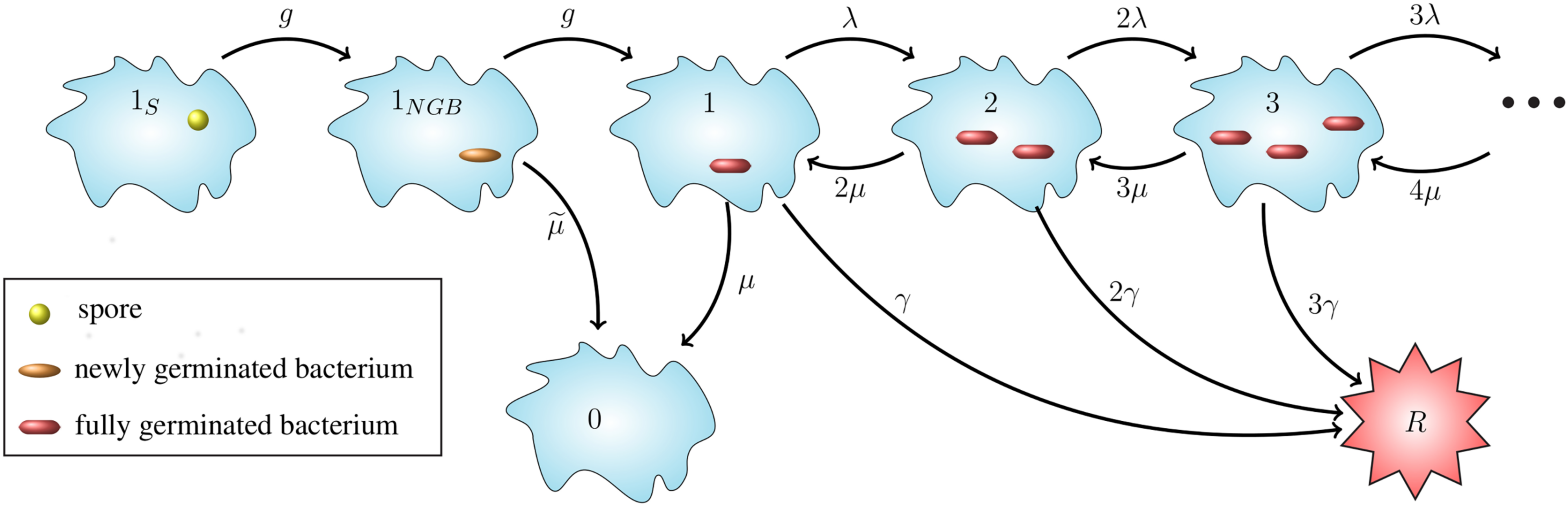
Intracellular infection model. State 1*_S_* represents a phagocytosed spore and state 1*_NGB_* a newly germinated bacterium (NGB). The germination rate from spore to NGB, and also the maturation rate from NGB to vegetative bacterium for a given spore is denoted by *g hours* ^-1^, which leads to an Erlang(2, *g*) distribution for the germination-maturation time. The rate *g* is assumed to vary between spores. The death rate of the newly germinated bacterium is given by $\tilde{\mu }$ *hours^−1^*. States *i* ∈ ℕ ∪ {0} represent *i* intracellular bacteria. State 0 represents recovery and state *R* the rupture of the cell. 0 and *R* are absorbing states for the stochastic process. Transitions between states *i* ∈ ℕ represent three types of events: transition to state *i* + 1 (division of a bacterium), to state *i* - 1 (death of a bacterium), and to state *R* (rupture of the host cell with release of *i* bacteria). The per bacterium division, death, and rupture rates are λ > 0, µ > 0 and γ > 0, respectively, all with units (*bacteria · hours*)^-1^, leading to a linear birth-and-death process with catastrophe. The infected cell survives for as long as it does not reach state *R*.

In this section we introduce a stochastic model for the dynamics of spores and bacteria in a single infected phagocyte, starting at the time when the cell phagocytoses a spore, and ending either with rupture and death of the cell and the release of bacteria into the extracellular environment, or with recovery of the cell and the elimination of any intracellular spores or bacteria. When considering low dose exposures, for which the multiplicity of infection (MOI) will be low, it is reasonable to assume that each phagocyte will only engulf at most one spore. Therefore, in what follows we only consider infection of a cell that has phagocytosed a single spore. The model presented here includes germination of the spore into a newly germinated bacterium, maturation of the newly germinated bacterium into a vegetative bacterium, replication of vegetative bacteria, death of bacteria, and rupturing of the host cell to release the intracellular bacteria (see **Figure 1**).

Our intracellular infection model, depicted in **Figure 1**, corresponds to a continuous-time Markov chain (CTMC), *χ* = {*X*(*t*), *t* ≥ 0}, taking values in the state-space *𝒮* = {1*_S_*, 1*_NGB_*, 0,1,2,…} ∪ {*R*}, where:

- 1*_S_* corresponds to the state where the host cell contains a spore and no bacteria,
- 1*_NGB_* corresponds to the state where the host cell contains a single newly germinated bacterium,
- {0,1,2, ... } represent the number of vegetative bacteria inside the host cell, so that 0 corresponds to the cell’s recovery, and
- *R* corresponds to the state of the host cell having ruptured.

Time *t* = 0 is the time at which the spore is phagocytosed, so the initial state of the process is assumed to be *X*(0) = 1*_S_*. Experimental work using murine macrophages has indicated that the ungerminated spore form of anthrax is not easily eliminated by host cells (23), so we assume that the initial spore will not die or be killed by the cell. We represent the germination-maturation time as an Erlang(2, *g*) distribution, as depicted in **Figure 1**, where the rate *g* has units *hours* ^-1^. This Erlang(2, *g*) representation is the simplest approach to consider a non-exponential distribution for the time that it takes the spore to become a vegetative bacterium, while including an intermediate, susceptible state as done in (15), and keeping the process Markovian. During the germination process, there is an increase in the hydration of the spore core and the spore cortex is broken down (1). These changes mean that the spore loses some of its resistance against the anti-microbial environment within the host cell and may be killed by the reactive oxygen and nitrogen species, and anti-microbial peptides within the phagolysosome (13). As a result, modelling the germination-maturation process with two stages allows one to incorporate this loss of resistance into the intermediate state 1*_NGB_*. We refer the reader to the *Discussion* section for some additional considerations on the Erlang(2, *g*) choice. The newly germinated bacterium can be killed by the cell, with rate $\tilde{\mu }\;hours^{-1}$, but cannot replicate unless it matures into a vegetative bacterium, represented by state 1. If the stochastic process reaches state 1, the subsequent replication of bacteria, death of bacteria, and rupture of the host cell is modelled as a birth-and-death process with catastrophe (24), with state-space {0,1,2, ... } ∪ {*R*} (see **Figure 1**). We introduce the per bacterium division, death and rupture rates λ, *µ* and γ, respectively, all with units (*bacteria · hours*)^-1^. The stochastic process has two absorbing states: the recovery state, 0, representing elimination of any intracellular spores and bacteria, and the rupture state, *R*, representing rupture of the infected cell and release of its entire content of bacteria.

For a CTMC, *χ*, the transition probability from state *i* ∈ *𝒮* to state *j* ∈ *𝒮* in time *t* is defined by,

(1) $$p_{i,j}(t)=\mathbb{P}(X(t)=j|X(0)=i).$$

We are interested in the dynamics of the process when the initial state is *X*(0) = 1*_S_*, representing an intracellular spore which was just phagocytosed. Therefore, when the initial state is *i* = 1*_S_*, we will omit the first index in the notation, so that the probability that the process is in state *j* at time *t*, given that it started with one spore, is denoted by

(2) $$p_{j}(t)=\mathbb{P}(X(t)=j|X(0)=1_{S}).$$

### Spore Germination

To describe the dynamics of the germination-maturation process, one can consider the transient state probabilities, *p_j_*(*t*), for each of the states, *j* ∈ {1*_S_*, 1*_NGB_*}. These probabilities obey the following differential equations, with initial conditions $p_{1_{S}}(0)=1\text{ and}p_{1_{NGB}}(0)=0$,

(3) $$\frac{dp_{1_{S}}}{dt}=-gp_{1_{S}},$$

(4) $$\frac{dp_{1_{NGB}}}{dt}=gp_{1_{S}}-(g+\tilde{\mu })p_{\;}1_{NGB}.$$

If a phagocyte engulfs a spore with germination rate *g* at time *t* = 0, then the cell will contain one spore at time *t* if *X*(*t*) = 1*_S_*, and zero spores if *X*(*t*) ≠ 1*_S_*. Hence, the mean number of intracellular spores at time *t* is equal to $p_{1_{S}}(t)$. Similarly, the mean number of intracellular newly germinated bacteria at time *t* is equal to $p_{1_{NGB}}(t)$. These are given by the solutions to Eqs. (3) and (4),

(5) $$p_{1_{S}}(t)=e^{-gt},$$

(6) $$p_{1_{NGB}}(t)=\frac{g}{\tilde{\mu }}\left(e^{-gt}-e^{-(g+\tilde{\mu })t}\right),$$

for any *t* ≥ 0. For each spore, we assume that its germination rate is equal to its maturation rate, and denote it by *g*. However, in order to reflect the heterogeneity in spore germination times as reported in the literature (1, 20, 21), we assume that the germination rate varies between spores. We consider here two different distributions for the germination rate and will explore and compare these two hypotheses in the *Parameter Calibration* section.

#### Continuous Gaussian Distribution

First, we assume that the germination rate varies continuously among spores, according to a normal distribution on the positive axis. In this case, the germination rate *g* for a given spore is a realisation of the continuous random variable $G∼N_{\left(0,+\infty \right)}\left(\mu_{g},\sigma_{g}^{2}\right)$, which is normally distributed with mean *μ_g_ hours* ^-1^ and standard deviation σ*_g_ hours* ^-1^, and is truncated to the interval (0,+∞). Therefore, the germination rate has probability density function given by

(7) $$f_{G}(g)=\frac{1}{Z}\frac{1}{\sigma_{g}\sqrt{2\pi }}e^{-\frac{1}{2}{\left(\frac{g-\mu_{g}}{\sigma_{g}}\right)}^{2}},\text{ for }g\in \left(0,+\infty \right),$$

where $Z=\Phi \left(\frac{\mu_{g}}{\sigma_{g}}\right)$ is a normalisation factor, and Φ is the cumulative distribution function of the standard normal distribution. Given this truncated normal distribution for the germination rate, the mean number of spores inside the infected cell at time *t* ≥ 0 is given by

(8) $$S(t)=\int_{0}^{+\infty }f_{G}(g)p_{1_{S}}(t)dg=\int_{0}^{+\infty }f_{G}(g)e^{-gt}dg=\frac{1}{Z}\exp\left(\frac{\sigma_{g}^{2}t^{2}}{2}-\mu_{g}t\right)\Phi \left(\frac{\mu_{g}-\sigma_{g}^{2}t}{\sigma_{g}}\right).$$

Similarly, the mean number of newly germinated bacteria inside the infected cell at time *t* ≥ 0 is given by

(9) $$B_{NGB}(t)=\int_{0}^{+\infty }f_{G}(g)p_{1_{NGB}}(t)dg=\int_{0}^{+\infty }f_{G}(g)\frac{g}{\tilde{\mu }}\left(e^{-gt}-e^{-\left(g+\tilde{\mu }\right)t}\right)dg=\frac{1}{\tilde{\mu }Z}\left(1-e^{-\tilde{\mu }t}\right)\left[\frac{\sigma_{g}}{\sqrt{2\pi }}e^{-\frac{\mu_{g}^{2}}{2\sigma_{g}^{2}}}+\left(\mu_{g}-\sigma_{g}^{2}t\right)\exp\left(\frac{\sigma_{g}^{2}t^{2}}{2}-\mu_{g}t\right)\Phi \left(\frac{\mu_{g}-\sigma_{g}^{2}t}{\sigma_{g}}\right)\right].$$

#### Discrete Bernoulli Distribution

Setlow in (20, 21) explains that some spores can be described as superdormant because they have very low germinant receptor levels and germinate extremely slowly, taking many hours or even days. Therefore, we propose a second choice for the germination rate, which assumes that the population of spores can be roughly split into two discrete groups, type A and type B, with type A having a faster germination rate than type B. Here, type A corresponds to the spores with “typical” germinant receptor levels, and type B corresponds to spores with significantly lower levels. We represent this as a discrete Bernoulli distribution with probability mass function, as follows

(10) $$f_{B}(g)=\{\begin{array}{ll} \varepsilon & if\;g=g_{A},\\ 1-\varepsilon & if\;g=g_{B}, \end{array}$$

for some parameter values *g_A_*> *g_B_* and *ε* ∈ (0,1). For this Bernoulli distribution, the mean number of spores and newly germinated bacteria inside the infected cell at time *t* ≥ 0 are, respectively,

(11) $$S(t)=\varepsilon e^{-g_{A}t}+\left(1-\varepsilon \right)e^{-g_{B}t},$$

(12) $$B_{NGB}(t)=\frac{\varepsilon g_{A}}{\tilde{\mu }}\left[e^{-g_{A}t}-e^{-\left(g_{A}+\tilde{\mu }\right)t}\right]+\frac{\left(1-\varepsilon \right)g_{B}}{\tilde{\mu }}\left(e^{-g_{B}t}-e^{-\left(g_{B}+\tilde{\mu }\right)t}\right).$$

### Cellular Fate: Rupture or Recovery

The CTMC in **Figure 1** has two absorbing states, *R* and 0, which denote rupture and recovery of the host cell, respectively. In this section we show how to compute the probability of either rupture or recovery of the cell, and the probability density functions of the recovery and rupture times. We also explain how to compute the conditional mean times taken to reach each of the two cellular fates. Finally, we show that the mean number of vegetative bacteria in the infected cell over time is proportional to the probability density function of the rupture time.

#### Probabilities and Times to Reach Absorbing States

The transient probabilities for the states *j* ∈ ℕ ∪ {0,R}, obey the following system of differential equations

(13) $$\frac{dp_{1}}{dt}=gp_{1_{NGB}}-\left(\lambda +\mu +\gamma \right)p_{1}+2\mu p_{2},$$

(14) $$\frac{dp_{j}}{dt}=\lambda (j-1)p_{j-1}+\mu (j+1)p_{j+1}-(\lambda +\mu +\gamma )jp_{j},\;j\geq 2,$$

(15) $$\frac{dp_{0}}{dt}=\tilde{\mu }p_{1_{NGB}}+\mu p_{1},$$

(16) $$\frac{dp_{R}}{dt}=\sum_{j=1}^{+\infty }\gamma jp_{j}.$$

The long-term probabilities of recovery or rupture for an infected cell, starting from any state *i* ∈ 𝒮 can be denoted, respectively, by

(17) $$r_{i}^{0}=\underset{t\to +\infty }{\lim}p_{i,0}(t),\;r_{i}^{R}=\underset{t\to +\infty }{\lim}p_{i,R}(t).$$

These probabilities can also be expressed in terms of the times it takes the process *χ* to reach states 0 and *R*, respectively. In particular, one can denote the times taken for the process to travel from state *i* to states 0 or *R*, respectively, by

(18) $$T_{i}^{0}=\inf\left\{t\geq 0:X(t)=0|X(0)=i\right\},\;T_{i}^{R}=\inf\{t\geq 0:X(t)=R|X(0)=i\}.$$

Since there is a choice of two possible absorbing fates (recovery or rupture), the random variables $T_{i}^{0}\text{ and }T_{i}^{R}$ may be infinite with non-zero probability. That is, the time to recovery, $T_{i}^{0}$, will be infinite if the process ends in the rupture state, and vice versa. Thus, we can write

(19) $$p_{i,0}(t)=\mathbb{P}(T_{i}^{0}\leq t),\;p_{i,ℛ}(t)=\mathbb{P}(T_{i}^{R}\leq t),$$

and

(20) $$r_{i}^{0}\;=\;\mathbb{P}(T_{i}^{0}<+\infty )=1-\mathbb{P}(T_{i}^{R}<+\infty )=1-r_{i}^{R}.$$

Carruthers et al. in (16) studied a very similar process to the one described here, for the non-sporulating bacteria *F. tularensis*. In this section we use some of their results. For instance, survival analysis allowed Carruthers et al. to show that the probability density function of the random variable $T_{1}^{R}$ (the rupture time starting from state 1, representing a single fully vegetative bacterium), is given by

(21) $$f_{T_{1}^{R}}(t)=\frac{\gamma {\left(b-a\right)}^{2}e^{-\lambda (b-a)t}}{{\left[b-1+(1-a)e^{-\lambda (b-a)t}\right]}^{2}},\;t\geq 0,$$

with

(22) $$a=\frac{(\lambda +\mu +\gamma )-\sqrt{{\left(\lambda +\mu +\gamma \right)}^{2}-4\mu \lambda }}{2\lambda },b=\frac{(\lambda +\mu +\gamma )+\sqrt{{\left(\lambda +\mu +\gamma \right)}^{2}-4\mu \lambda }}{2\lambda }.$$

They also showed that the probability that a cell eventually ruptures, starting in state 1 with one vegetative bacterium, is $r_{1}^{R}=1-a$, and the probability that a cell eventually recovers is $r_{1}^{0}=a$. One can adapt these results to our model with the help of first-step analysis, to find the probabilities of rupture and recovery starting with one initial spore in state 1*_S_*. If a phagocyte is infected with a spore at time *t* = 0, then at some time point, the initial spore will germinate, transitioning to the intermediate state of newly germinated bacterium (NGB). At some later time, the NGB will either die, with probability $\tilde{\mu }/(\tilde{\mu }+g)$, or will mature into a vegetative bacterium with probability $g/(\tilde{\mu }+g)$. Since it is certain that the first step of the process will be the transition from spore to newly germinated bacterium, the probability of eventual recovery or rupture starting from state 1*_S_* is the same as the probability of recovery or rupture starting from state 1*_NGB_*. The only way that the process will eventually reach the rupture state is if the newly germinated bacterium matures into a vegetative bacterium, and then the cell eventually ruptures starting from state 1. On the other hand, the cell can recover if either the newly germinated bacterium dies before it matures into a vegetative bacterium, or the newly germinated bacterium matures and the cell eventually recovers starting from state 1. In particular, given a germination rate *g* for the initial spore, the probabilities for rupture and recovery, starting from state 1*_S_*, are given by

(23) $$r_{1_{S}}^{R}(g)=r_{1_{NGB}}^{R}(g)=\frac{g}{\tilde{\mu }+g}\;r_{1}^{R}=\frac{g(1-a)}{\tilde{\mu }+g},r_{1_{S}}^{0}(g)=r_{1_{NGB}}^{0}(g)=\frac{\tilde{\mu }}{\tilde{\mu }+g}+\frac{g}{\tilde{\mu }+g}\;r_{1}^{0}=\frac{\tilde{\mu }+ga}{\tilde{\mu }+g}.$$

One can also derive the probability density functions of the time to rupture and the time to recovery. These shed light on the distribution of times to rupture and recovery across cells that have been infected with one spore. In what follows, we denote the random variable for the time to transition from state *i* to state *j* by $T_{i}^{j}=\inf\{t\geq 0:X(t)=j|X(0)=i\}$, and the probability density function for this random variable is denoted by $f_{T_{i}^{j}}(t)$.

First, we consider the total time for the initial intracellular spore to germinate and mature into a vegetative bacterium, given by the random variable $T_{1_{S}}^{1}$, with probability density function, $f_{T_{1_{S}}^{1}}(t)$. This function will be needed later to calculate the probability density function of the times to rupture and recovery. To find the function $f_{T_{1_{S}}^{1}}(t)$, let $F_{T_{1_{S}}^{1}}(t)=\mathbb{P}(T_{1_{S}}^{1}\leq t)$ be the probability that the cell contains an intracellular vegetative bacterium by time *t*, given that the germination rate of the initial spore is equal to *g*. Let us consider a small time step Δ*t*, such that only one transition can occur in the interval (*t*,*t* + Δ*t*). If the cell has not entered state 1, representing a vegetative bacterium, before time *t*, then it will only be possible to enter state 1 before time *t* + Δ*t* if the cell is in state 1*_NGB_* at time *t*. Furthermore, if the phagocyte contains a newly germinated bacterium at time *t*, then the probability to transition to a vegetative bacterium between time *t* and *t* + Δ*t* is *g*Δ*t*. Hence,

(24) $$F_{T_{1_{S}}^{1}}(t+\Delta t)=F_{T_{1_{S}}^{1}}(t)+p_{1_{NGB}}(t)g\Delta t.$$

The function $F_{T_{1_{S}}^{1}}(t)$ is the cumulative distribution function of the random variable, $T_{1_{S}}^{1}$, for the time that the process takes to reach a vegetative bacterium, starting from one spore. Therefore, the probability density function for this random variable is

(25) $$f_{T_{1_{S}}^{1}}(t;g)=\frac{dF_{T_{1_{S}}^{1}}(t)}{dt}=g\;p_{1_{NGB}}(t)=\frac{g^{2}}{\tilde{\mu }}(e^{-gt}-e^{-(g+\tilde{\mu })t}),\;t\geq 0,$$

where we have written explicitly that $f_{T_{1_{S}}^{1}}$ is a function of the germination rate *g*. It can be verified that

(26) $$\mathbb{P}(T_{1_{S}}^{1}<+\infty )=\int_{0}^{+\infty }f_{T_{1_{S}}^{1}}(t;g)dt=\frac{g}{g+\tilde{\mu }},$$

which is the probability that the process will eventually reach state 1, or equivalently, the probability that a spore will mature into a vegetative bacterium instead of being cleared by the infected cell.

##### Time to Recovery

Here we show how to compute the probability density function, $f_{T_{1_{S}}^{0}}(t)$, for the time to recovery of an infected cell starting with one spore. Following the same approach as above, the probability density function for the random variable $T_{1_{S}}^{0}$, given that the spore has germination rate *g*, is

(27) $$f_{T_{1_{S}}^{0}}(t;g)=\frac{dF_{T_{1_{S}}^{0}}(t)}{dt}=\tilde{\mu }p_{1_{NGB}}(t)+\mu p_{1}(t)=\;g(e^{-gt}-e^{-(g+\tilde{\mu })t})+\mu \int_{0}^{t}f_{T_{1_{S}}^{1}}(s;g)p_{1,1}(t-s)ds,\;t\geq 0,$$

where *p*_1,1_(*t*) is the probability that the process, starting in state 1, is in state 1 at time t, and can be derived from results in (16) as follows,

(28) $$p_{1,1}(t)=\frac{{\left(a-b\right)}^{2}e^{-\lambda (b-a)t}}{{\left(ae^{-\lambda (b-a)t}-b\right)}^{2}},$$

with *a* and *b* defined in Eq. (22). We note that the probability density function for the time to recovery from state 1 can be written as

(29) $$f_{T_{1}^{0}}(t)=\mu p_{1,1}(t).$$

When the germination rate follows a continuous Gaussian distribution (see Eq. (7)), the probability density of the recovery time starting from state 1*_S_* is given by

(30) $$f_{T_{1_{S}}^{0}}(t)=\int_{0}^{+\infty }f_{G}(g)f_{T_{1_{S}}^{0}}(t;g)dg.$$

Alternatively, if the germination rate follows a discrete Bernoulli distribution (see Eq. (10)), the probability density of recovery times is given by

(31) $$f_{T_{1_{S}}^{0}}(t)=\varepsilon f_{T_{1_{S}}^{0}}(t;g_{A})+(1-\varepsilon )f_{T_{1_{S}}^{0}}(t;g_{B}).$$

The probability density of recovery times yields the distribution of recovery times across cells, since each phagocyte is assumed to be initially infected by a single spore. To gain insights into the expected time of recovery, one can also compute the conditional mean time to recovery of an infected cell, which is the expected time to recovery, given that the cell eventually recovers. This is denoted by $E[T_{1_{S}}^{0}|T_{1_{S}}^{0}<+\infty ]$, given that the eventual recovery of a cell is equivalent to its recovery time being finite. For any initial state *i* ∈ *𝒮*, one can define the restricted mean time to recovery as $\tau_{i}^{0}=E[T_{i}^{0}\cdot \delta_{T_{i}^{0}<+\infty }],$ where δ*_A_* is equal to 1 if *A* is satisfied and 0 otherwise. Then the conditional mean time to recovery, starting from state *i* ∈ *𝒮*, is defined by

(32) $$E[T_{i}^{0}|T_{i}^{0}<+\infty ]=\frac{\tau_{i}^{0}}{r_{i}^{0}}.$$

Hence, in order to calculate the conditional expectation, $E[T_{1_{S}}^{0}|T_{1_{S}}^{0}<+\infty ]$, one must restrict the sample space of $T_{1_{S}}^{0}$ to finite values, and divide by the probability that the recovery time is finite. The set of finite recovery times can be partitioned into the set where $T_{1_{S}}^{1}=+\infty$ and the set where $T_{1_{S}}^{1}<+\infty$. In other words, the cell can either recover without ever containing vegetative bacteria, or the cell can recover after having contained at least one vegetative bacterium. Therefore, the restricted mean time to recovery can be written as follows

$$\tau_{1_{S}}^{0}=E[T_{1_{S}}^{0}\cdot \delta_{T_{1_{S}}^{1}=+\infty }]+E[T_{1_{S}}^{0}\cdot \delta_{T_{1_{S}}^{1}<+\infty }\cdot \delta_{T_{1}^{0}<+\infty }].$$

Using the fact that $T_{1_{S}}^{0}\cdot \delta_{T_{1_{S}}^{1}<+\infty }\cdot \delta_{T_{1}^{0}<+\infty }=(T_{1_{S}}^{1}+T_{1}^{0})\cdot \delta_{T_{1_{S}}^{1}<+\infty }\cdot \delta_{T_{1}^{0}<+\infty }$, and that $T_{1_{S}}^{1}\text{ and }T_{1}^{0}$ are independent, one finds that the restricted mean time to recovery for a cell infected with one spore with germination rate *g*, is

(33) $$\tau_{1_{S}}^{0}(g)=\left(\frac{1}{g}+\frac{1}{\tilde{\mu }+g}\right)\frac{\tilde{\mu }}{\tilde{\mu }+g}+E[T_{1_{S}}^{1}\cdot \delta_{T_{1_{S}}^{1}<+\infty }]\mathbb{P}(T_{1}^{0}<+\infty )+E[T_{1}^{0}\cdot \delta_{T_{1}^{0}<+\infty }]\mathbb{P}(T_{1_{S}}^{1}<+\infty )=\frac{\tilde{\mu }(\tilde{\mu }+2g)}{g{\left(\tilde{\mu }+g\right)}^{2}}+a\int_{0}^{+\infty }tf_{T_{1_{S}}^{1}}(t;g)dt+\frac{g}{g+\tilde{\mu }}\int_{0}^{+\infty }tf_{T_{1}^{0}}(t)\;dt=\frac{g}{\tilde{\mu }+g}[\frac{\left(\tilde{\mu }+2g)(ga+\tilde{\mu }\right)}{g^{2}(\tilde{\mu }+g)}+\frac{1}{\lambda }\log(\frac{b}{b-a})],$$

where we have made use of Eqs. (25), (26), and (29). The values *a* and *b* are defined in Eq. (22).

With this restricted mean time at hand, and the probability of recovery in Eq. (23), one can use Eq. (32) to find the conditional mean time until recovery for the two different distributions of the germination rate. In particular, when the germination rate follows a continuous Gaussian distribution in Eq. (7), the conditional mean time to recovery for an infected cell starting with one spore is given by

(34) $$E[T_{1_{S}}^{0}|T_{1_{S}}^{0}<+\infty ]=\frac{\int_{0}^{+\infty }f_{G}(g)\tau_{1_{S}}^{0}(g)\;dg}{\int_{0}^{+\infty }f_{G}(g)r_{1_{S}}^{0}(g)\;dg}.$$

Alternatively, if one considers the discrete Bernoulli distribution for the germination rate, the conditional mean time to recovery is given by

(35) $$E[T_{1_{S}}^{0}|T_{1_{S}}^{0}<+\infty ]=\frac{\varepsilon \tau_{1_{S}}^{0}(g_{A})+(1-\varepsilon )\tau_{1_{S}}^{0}(g_{B})}{\varepsilon r_{1_{S}}^{0}(g_{A})+(1-\varepsilon )r_{1_{S}}^{0}(g_{B})}.$$

##### Time to Rupture

The time taken for the initial phagocytosed spore to transition into a vegetative bacterium is given by the random variable $T_{1_{S}}^{1}$ and the time from vegetative bacterium to rupture is denoted by $T_{1}^{R}$. Thus, the total time between the cell engulfing a spore, and the time of rupture, is $T_{1_{S}}^{R}=T_{1_{S}}^{1}+T_{1}^{R}$. The corresponding probability density function for $T_{1_{S}}^{1}$ was given by $f_{T_{1_{S}}^{1}}(t;g)$ in Eq. (25), and the probability density function for the rupture time starting from one vegetative bacterium was given by $f_{T_{1}^{R}}(t)$ in Eq. (21). One can convolve these two functions to find the probability density function for the total time to rupture, giving 

(36) $$f_{T_{1_{S}}^{R}}(t;g)=\int_{0}^{t}f_{T_{1_{S}}^{1}}(s;g)f_{T_{1}^{R}}(t-s)\;ds.$$

When the germination rate across spores follows the continuous Gaussian distribution, the density of rupture times is given by

(37) $$f_{T_{1_{S}}^{R}}(t)=\int_{0}^{+\infty }f_{G}(g)f_{T_{1_{S}}^{R}}(t;g)\;dg.$$

Alternatively, in the discrete Bernoulli case, the density of rupture times is given by

(38) $$f_{T_{1_{S}}^{R}}(t)=\varepsilon f_{T_{1_{S}}^{R}}(t;g_{A})+(1-\varepsilon )f_{T_{1_{S}}^{R}}(t;g_{B}).$$

As done previously for recovery, one can also calculate the conditional mean time to rupture, denoted $E[T_{1_{S}}^{R}|T_{1_{S}}^{R}<+\infty ]$ and defined similarly to Eq. (32) with 0 replaced by *R*. Since the random variables $T_{1_{S}}^{1}\text{ and }T_{1}^{R}$ are independent, it can be shown that the restricted mean time to rupture, for a cell initially infected with a spore with germination rate *g*, is

(39) $$\tau_{1_{S}}^{R}(g)=E[T_{1_{S}}^{1}\cdot \delta_{T_{1_{\text{s}}}^{1}<+\infty }]\mathbb{P}(T_{1}^{R}<+\infty )+E[T_{1}^{R}\cdot \delta_{T_{1}^{R}<+\infty }]\mathbb{P}(T_{1_{\text{s}}}^{1}<+\infty )=(1-a)\int_{0}^{+\infty }tf_{T_{1_{S}}^{1}}(t;g)\;dt+\frac{g}{g+\tilde{\mu }}\int_{0}^{+\infty }tf_{T_{1}^{R}}(t)\;dt=\frac{g}{\tilde{\mu }+g}[\frac{\left(\tilde{\mu }+2g)(1-a\right)}{g(\tilde{\mu }+g)}+\frac{1}{\lambda }\log(\frac{b-a}{b-1})],$$

where we have made use of Eqs. (25), (26) and (21). The values *a* and *b* are defined in Eq. (22).

Given this restricted mean time, and the probability of rupture from Eq. (23), one can use Eq. (32), with 0 replaced by *R*, to find the conditional mean time until rupture, for the two different distributions of the germination rate. In particular, when the germination rate across spores follows a continuous Gaussian distribution, the conditional mean time to rupture is of the same form as Eq. (34), with 0 replaced by *R*. Similarly, if one considers the discrete Bernoulli distribution for the germination rate, the conditional mean time to rupture is of the same form as Eq. (35), with 0 replaced by *R*.

#### Number of Intracellular Vegetative Bacteria

Given a particular germination rate *g* for the phagocytosed spore, we denote the mean number of intracellular vegetative bacteria at time *t* by *B*_ν_(*t*; *g*), where

(40) $$B_{v}(t;g)=\sum_{j=1}^{+\infty }j\;p_{j}(t)=\frac{1}{\gamma }\frac{dp_{R}(t)}{dt},$$

with the second equality arising from Eq. (16). Since *p_R_*(*t*) represents the cumulative distribution function of the rupture time starting with one spore, this means that the average number of vegetative bacteria is proportional to the probability density function of the rupture time. That is, the mean number of vegetative bacteria at time *t*, given germination rate *g*, is

(41) $$B_{v}(t;g)=\frac{f_{T_{1_{S}}^{R}}(t;g)}{\gamma }.$$

Once this is averaged over the possible values of the germination rate, *g*, for either germination rate distribution hypothesis, the mean number of vegetative bacteria inside a cell at time *t*, is given by

(42) $$B_{v}(t)=\frac{f_{T_{1_{S}}^{R}}(t)}{\gamma },$$

where$\;f_{T_{1_{S}}^{R}}(t)$ is defined in Eqs. (37) and (38) for the two germination rate distributions.

### Rupture Size Distribution

For an infected cell described by the CTMC *χ* it is possible to find the probability distribution of its *rupture size*, which is the number of bacteria released into the extracellular environment from the infected cell. If the time for the process to enter state 0 is finite, then the rupture size is equal to 0, indicating that the host cell recovers and does not release any bacteria. On the other hand, if the time to reach state *R*, denoted by $T_{1_{S}}^{R}$, is finite, and $X(T_{1_{S}}^{R}-\Delta t)=n$ for small and positive Δ*t*, this means that the process transitions into the rupture state from state *n* ∈ ℕ. This corresponds to the death and rupture of the host cell, and the release of *n* bacteria into the extracellular environment. Let $R_{i}^{n}$ denote the probability that the cell will release *n* bacteria in total, given that the process starts at state *i* ∈ *𝒮*. This is defined as

(43) $$R_{i}^{n}=\{\mathbb{P}(T_{i}^{0}<+\infty ),for\;n=0,\mathbb{P}((T_{i}^{R}<+\infty )\text{ and }(X(T_{i}^{R}-\Delta t)=n)),for\;n\in \mathbb{N}.$$

With this definition, $R_{i}^{0}$ is the probability that the cell recovers, so $r_{i}^{0}=R_{i}^{0}$. For *n* ∈ ℕ, $R_{i}^{n}$ is the probability that the cell ruptures and releases *n* bacteria, so the overall probability of rupture is $r_{i}^{R}=\Sigma_{n=1}^{+\infty }R_{i}^{n}$. For states *i* ∈ ℕ, the probabilities $R_{i}^{n}$ do not depend on the germination rate, *g*. However for *i* ∈ {1*_S_*, 1*_NGB_*}, these probabilities do depend on the germination rate, and so will be denoted by $R_{1_{S}}^{n}(g)\text{ and }R_{1_{NGB}}^{n}(g)$.

We now follow the method of Karlin and Tavare from (24) to find the probabilities $R_{1}^{n}$. If the cell begins with a vegetative bacterium, so that *X*(0) = 1, then for a small time interval Δ*t* → 0, one has,

(44) $$\mathbb{P}((X(t)=n)\text{ and }(t<T_{1}^{R}\leq t+\Delta t))=\mathbb{P}(X(t)=n)\times \mathbb{P}(t<T_{1}^{R}\leq t+\Delta t\;|\;X(t)=n)=p_{1,n}(t)n\gamma \Delta t,$$

since if the cell contains *n* bacteria at time *t*, the probability of rupture between time *t* and *t* + Δ*t* is *n*γΔ*t*. An expression for *p*_1,_ *_n_*(*t*), which is the probability that a cell contains *n* bacteria at time *t*, given that it contains one bacterium at time 0, was given by Carruthers et al. in (16),

(45) $$p_{1,n}(t)=\frac{{\left(b-a\right)}^{2}e^{-\lambda (b-a)t}{\left(e^{-\lambda (b-a)t}-1\right)}^{n-1}}{{\left(ae^{-\lambda (b-a)t}-b\right)}^{n+1}},\;n\geq 1.$$

Hence for an infected phagocyte starting with one vegetative bacterium, the probability that the cell releases *n* ≥ 1 bacteria, $R_{1}^{n}$, is then

(46) $$R_{1}^{n}=\int_{0}^{+\infty }p_{1,n}(t)n\gamma \;dt=\frac{\left(1-a)(b-1\right)}{b^{n}},$$

with *a* and *b* defined in Eq. (22). Moreover, a first-step argument allows one to obtain the probability $R_{1_{S}}^{n}(g)\text{from}R_{1}^{n}$. For *n* ≥ 1 and germination rate *g*, one has

(47) $$R_{1_{S}}^{n}(g)=R_{1_{NGB}}^{n}(g)=\frac{g}{\tilde{\mu }+g}R_{1}^{n}.$$

When the germination rate across spores follows the continuous Gaussian distribution, the probability that the rupture size of a cell initially infected with one spore is equal to *n* ∈ ℕ bacteria, is given by

(48) $$R_{1_{S}}^{n}=R_{1}^{n}\int_{0}^{+\infty }f_{G}(g)\frac{g}{\tilde{\mu }+g}dg.$$

Alternatively, if one considers the discrete Bernoulli distribution for the germination rate, the probability that the rupture size of a cell initially infected with one spore is equal to *n* ∈ ℕ bacteria, is given by

(49) $$R_{1_{S}}^{n}=R_{1}^{n}(\frac{\varepsilon g_{A}}{\tilde{\mu }+g_{A}}+\frac{(1-\varepsilon )g_{B}}{\tilde{\mu }+g_{B}}).$$

## Parameter Calibration

In this section we make use of experimental data from an *in vitro* study by Kang et al. (23), which was discussed by Pantha et al. in (15) and used to calibrate their ODE model. In the experiment 10^6^ murine peritoneal macrophages were incubated with different numbers of Sterne 34F2 strain spores for 30 minutes, during which time phagocytosis occurred (23). The ratio of spores to cells in the solution at the start of the incubation period is called the multiplicity of infection (MOI) and in this case the four MOIs considered were spore to macrophage ratios of 1:1, 1:2, 1:10 and 1:20, corresponding to the initial number of spores in the solution of 10^6^, 5 × 10^5^, 10^5^, and 5 × 10^4^. At the end of 30 minutes, the solutions were washed, so no extracellular spores remained, and no more spores were phagocytosed after this time. Then the solutions were incubated with an antibacterial agent called gentamicin for 30 minutes to remove any extracellular bacteria. At various time points after this, samples of parallel replicates of the experiment were washed and the number of intracellular spores and bacteria determined. The data from this experiment is given in (15, Tables 2 and 3). While in reality cells could phagocytose more than one spore each in this experiment, this is less likely to happen when the average number of spores per cell in the solution is low. Therefore we only use the data for MOI 1:2, 1:10 and 1:20 to perform the parameter calibration, since these low MOIs will be more consistent with our model assumption in the *Materials and Methods* section that each macrophage engulfs at most one spore, leading to the initial condition for our model in **Figure 1**. Still, once we have obtained posterior samples of the parameters, we will compare our model predictions to the MOI 1:1 data, as a qualitative validation.

The experiment described above was also performed using a germination-deficient strain of *B. anthracis* spores, in which spore germination is inhibited. The average spore counts at one hour from two duplicate samples that used the germination-deficient strain are provided in **Table 1**. The number of spores of the germination-deficient strain would have remained unchanged between 0.5 hours and 1 hour, because they will not have germinated, and all extracellular spores were removed by washing at 0.5 hours, so there would have been no more phagocytosis after this time. Thus, if one assumes that there is no difference in spore phagocytosis rates between the germination-deficient and Sterne strains, one concludes that the spore counts for the germination-deficient strain are a good representation of the total number of Sterne strain spores that would have been phagocytosed during the first 0.5 hours of the experiment for each MOI. In the parameter calibration for their Phase II subsystem model, Pantha et al. used these numbers of intracellular spores from the germination-deficient experiment as the initial condition for the intracellular dynamics. The justification given is that germination does not seem to be a dominating process at 0.5 hours, so the number of spores of the Sterne strain at 0.5 hours will be similar to the number of germination-deficient spores at the same time point. Therefore, we make here the same assumption that no germination of the Sterne strain spores has occurred before 0.5 hours. Because of this, our estimates for *g_A_* and *g_B_* in the discrete Bernoulli model should be interpreted with this 30-min delay in mind, and in particular our estimated germination rates might be slightly overestimated as a result. However, this delay could possibly be interpreted as a time-lag after phagocytosis for the activation of germination to occur. Furthermore, after learning about the parameters with ABC-SMC inference, we will see that even the spores with a quicker germination rate (type A) take on average longer than an hour to germinate, so it seems (*a posteriori*) reasonable to assume that germination does not happen in the first 30 minutes of the experiment.

**Table 1:** Data taken from (15, Table 2), giving the average number of intracellular spores of two replicates of the experiment counted at 1 hour when using spores of a germination-deficient strain of anthrax.

Intracellular germination-deficient spore count at one hour
MOI 1:1	377500
MOI 1:2	139000
MOI 1:10	30500
MOI 1:20	13925

**Figure 2:**
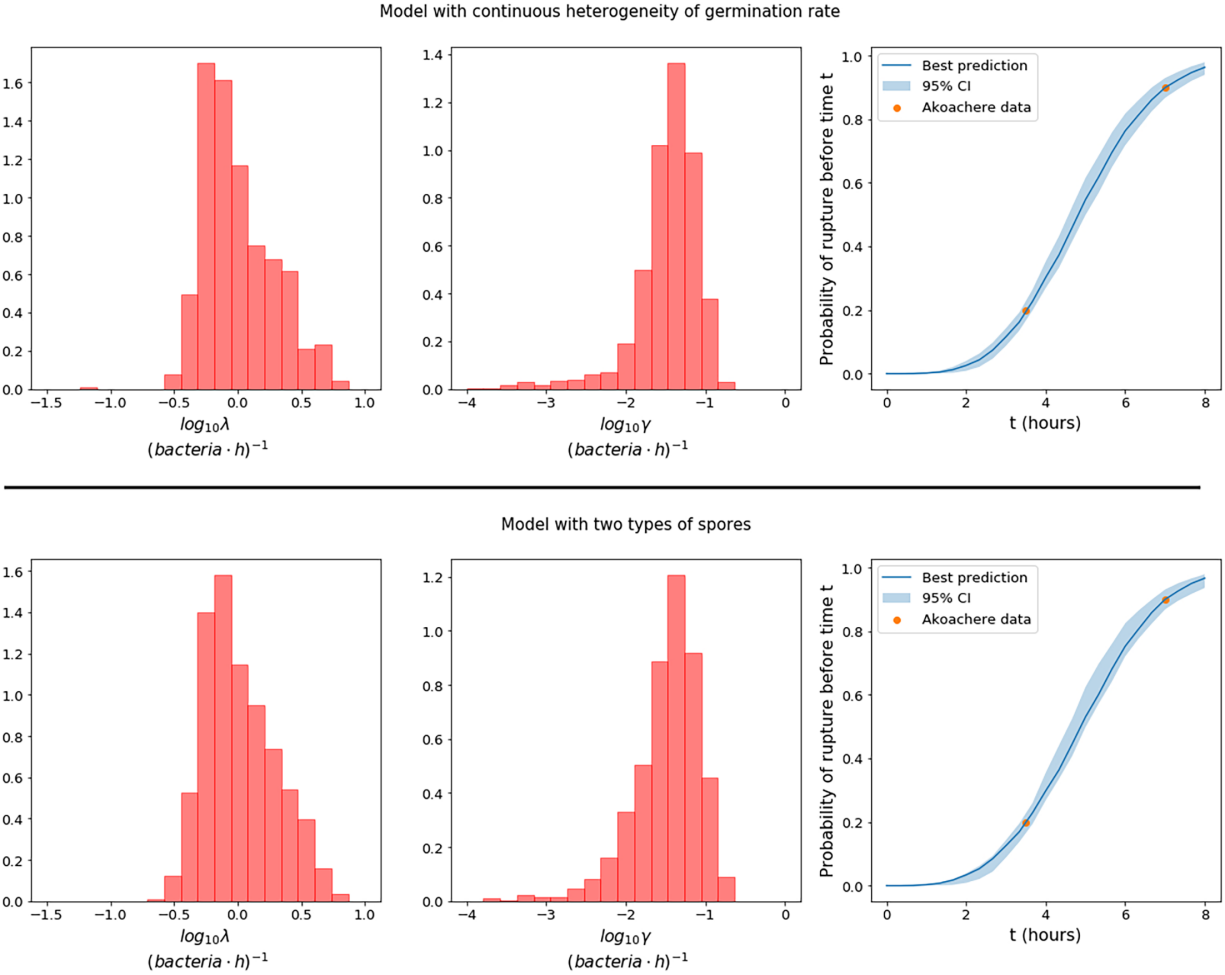
Prior distributions for the replication rate, λ, and the rupture rate γ, in the model with continuous heterogeneity of germination rate (top row), and the model with two types of spores (bottom row), obtained by using observations from Akoachere et al. (26) of the proportion of dead macrophages at two different time points. The plots on the right show the best predictions (solid line) of the fraction of cells that would be expected to rupture before time t in an experiment with MOI 20:1, compared to the data from Akoachere et al. (26). The pointwise 95% credible intervals (shaded region) are shown to represent the uncertainty in the parameter values.

The number of spores of the germination-deficient strain should have remained unchanged between 0.5 hours and 1 hour, because they cannot germinate, and all extracellular spores were removed by washing at 0.5 hours, so there would have been no more phagocytosis after this time. We note that the value for MOI 1:10 reported in (15, Table 2) was inconsistent with that observed in (15, Figure 2), so the second one is used here, since it is more consistent with the trajectory over time for the spore counts in (15, Figure 2) for MOI 1:10, and also so that our predictions are comparable with those in (15).

In the data from the experiment, time *t* = 0 corresponds to the start of the incubation period of cells and spores (23). On the other hand, our model considers a single host cell that begins with one intracellular spore, and *t* = 0 is assumed to be the start of the germination process of this spore. Since phagocytosis only occurs during the first 0.5 hours of the experiment, and we assume that germination does not occur until after the first 0.5 hours of the experiment, we do not explicitly include phagocytosis in our mathematical model. Instead, we modify the time points so that *t* = 0.5 in the experiment corresponds to *t* = 0 in our model. That is, we take the number of intracellular spores from the germination-deficient experiment, given in **Table 1**, to be our initial conditions for *t* = 0, and use the data as it is shown in **Table 2**.

**Table 2:** Data for the number of intracellular spores and bacteria present at different time points, which have been used to perform ABC-SMC.

	Time (hours)	0	0.5	2.5	4.5	23.5
MOI 1:2	Number of intracellular spores	139000	105000	11400	10250	29500
	Number of intracellular bacteria	0	23000	67100	52250	20000
MOI 1:10	Number of intracellular spores	30500	27000	12000	9100	2750
	Number of intracellular bacteria	0	9500	14000	7650	2500
MOI 1:20	Number of intracellular spores	13925	7900	6450	3100	300
	Number of intracellular bacteria	0	6000	2250	1750	1000

These data have been taken from (15, Tables 2 and 3), although time points are shifted by 30 min to account for the first phase of the experiment where phagocytosis occurs. The initial conditions (t = 0) correspond to the values reported in **Table 1** from the germination-deficient experiment. The counts at each time point are averages of two experimental replicates.

We have performed Approximate Bayesian Computation Sequential Monte Carlo (ABC-SMC) (22) to estimate our intracellular model parameters. This method involves choosing prior distributions for the model parameters and then carrying out multiple iterations of the ABC algorithm. At each iteration, parameter values are sampled from the posterior distribution of the previous iteration and are perturbed with a kernel function. Here we use a component-wise uniform perturbation kernel, so that each component of the parameter set is perturbed independently in a uniform interval. The perturbed parameter set is then used to obtain a model prediction and is accepted if the distance between the model and the data falls below the distance threshold for that iteration. We have used a sequence of decreasing distance thresholds, such that the distance threshold at each iteration is the median of the distances from the accepted parameter sets in the previous iteration. In this manner, one obtains a set of distributions for the parameters that converge to the posterior distribution. In the parameter calibration results that follow, we are considering a posterior sample of size 10^3^ from the final iteration of the ABC algorithm.

In this section we present the results obtained from using the MOI 1:2, 1:10 and 1:20 data sets to obtain posterior parameter distributions, first for the continuous model of germination rate, and then for the discrete Bernoulli model. The authors of (15) used these data to estimate different parameter sets for each MOI, mentioning that for lower MOIs the smaller average intracellular burden could give a better environment for spores to germinate and bacteria to replicate, leading to larger values of the parameters. However, since we are assuming that every infected cell begins with only a single intracellular spore, this means that the cellular MOI is assumed to be identical across all data sets, and in this way the MOI is simply a measure of the system size. Hence we believe that the parameters considered in our stochastic model may not depend on the initial conditions given by the MOI. Thus, we use the three data sets together to obtain a single set of estimates for the parameters, aiming to give a reasonable fit to the data sets with a significantly smaller number of parameters in our model.

In the experiment, the samples were washed each time before counting the number of intracellular spores and bacteria. Therefore, any bacteria released from a ruptured macrophage would be removed during the washing process and would not contribute to the number of bacteria observed in the data. Thus, to compare our model with the data, we use our model to calculate the per cell mean number of intracellular spores and bacteria over time. For the model with continuous distribution for the germination rate, Eqs. (8), (9), and (42) define the expected number of spores and bacteria in a cell at time *t*, given that it contained a spore at time *t* = 0. Since each infected cell is assumed to be independent, we can multiply these by the number of initial spores, to obtain the mean number of spores and bacteria present inside the population of cells at time *t*. Let *S*_0_ be the total number of initial intracellular spores for the population of cells at time *t* = 0. Then for the hypothesis of continuous heterogeneity in the germination rate, the total expected number of spores in all cells at time *t* is given by

(50) $$S^{*}(t)=\frac{S_{0}}{Z}\exp(\frac{\sigma_{g}^{2}t^{2}}{2}-\mu_{g}t)\Phi (\frac{\mu_{g}-\sigma_{g}^{2}t}{\sigma_{g}}).$$

The total (in all cells) expected number of intracellular bacteria at time *t* hours, including newly germinated bacteria and vegetative bacteria, is given by

(51) $$B^{*}(t)=S_{0}[\frac{1}{\tilde{\mu }Z}(1-e^{-\tilde{\mu }t})(\frac{\sigma_{g}}{\sqrt{2\pi }}e^{-\frac{\mu_{g}^{2}}{2\sigma_{g}^{2}}}+(\mu_{g}-\sigma_{g}^{2}t)\exp\;(\frac{\sigma_{g}^{2}t^{2}}{2}-\mu_{g}t)\Phi (\frac{\mu_{g}-\sigma_{g}^{2}t}{\sigma_{g}}))+B_{v}(t)]$$

where *B*_ν_(*t*) is the expected number of vegetative bacteria in one cell at time *t*, defined in Eq. (42). Similarly, for the model with two discrete germination rates, the model predictions for number of spores and bacteria are given by

(52) $$S^{*}(t)=S_{0}[\varepsilon e^{-g_{A}t}+(1-\varepsilon )e^{-g_{B}t}],$$

and

(53) $$B^{*}(t)=S_{0}\left[\frac{\varepsilon g_{A}}{\tilde{\mu }}\left(e^{-g_{A}t}-e^{-(g_{A}+\tilde{\mu })t}\right)+\frac{(1-\varepsilon )g_{B}}{\tilde{\mu }}\left(e^{-g_{B}t}-e^{-(g_{B}+\tilde{\mu })t}\right)+B_{v}(t)\right],$$

where *B*_ν_(*t*) is the expected number of vegetative bacteria in one cell at time *t*. In the ABC-SMC, we compare the data to the model outputs given by *S* ^∗^(*t*) and *B* ^∗^(*t*), with the initial number of spores equal to *S*_0_ = 139000 for MOI 1:2, *S*_0_ = 30500 for MOI 1:10 and *S*_0_ = 13925 for MOI 1:20.

In the model with continuous germination rate distribution, where the germination rate follows a truncated normal distribution, $G∼N_{\left(0,+\infty \right)}(\mu_{g,}\sigma_{g}^{2})$, the parameters characterising germination that we aim to estimate are the mean germination rate *µ_g_*, and its standard deviation σ*_g_*. In the discrete Bernoulli model, the parameters characterising germination that we aim to estimate are the probability *ε* that a given spore is of type A, and the two germination rates, *g_A_* and *g_B_*. The rest of the parameters that we wish to estimate are common to both versions of the model: the death rate of newly germinated bacteria, $\tilde{\mu }$, the replication rate of vegetative bacteria, λ, the death rate of vegetative bacteria, *µ*, and the rupture rate, γ. To calibrate these parameters, we performed ABC-SMC, and compared our model with the numbers of intracellular spores and bacteria over time from the experiment by Kang et al. (23). Similarly to (25), we make use of the Euclidean distance for the logarithm of the predicted values and observed data, given by

(54) $$d(model\;prediction,\;Data)=\sqrt{\sum_{i\in \{2,10,20\}}\sum_{t\in T}(\log(\frac{S_{i}^{*}(t)}{s_{i}(t)}){)}^{2}+(\log(\frac{B_{i}^{*}(t)}{b_{i}(t)}){)}^{2},}$$

where *T* = {0.5,2.5,4.5,23.5}, $S_{i}^{*}(t)\text{ and }B_{i}^{*}(t)$ are the respective model predictions for the number of spores and bacteria at time *t* for MOI 1:*i*, and *s_i_*(*t*), *b_i_*(*t*) are the respective observed number of spores and bacteria at time *t*, given by the data for MOI 1:*i*.

To perform ABC-SMC, one needs to choose prior distributions from which to sample the parameter values at the first iteration. To inform the selection of some of these prior distributions, we leverage data from Akoachere et al. (26), who observed that after infecting murine macrophages with *B. anthracis* Sterne strain spores at a spore to macrophage ratio (MOI) of 20:1, 20% of cells had ruptured by 3.5 hours, and 90% had ruptured by 7 hours. We preliminarily fit our model to these data and use the results to estimate a potential prior distribution for the replication and rupture rates, λ and γ. We are unable to learn much about the other model parameters in this way, since only two data points based on a single experiment are available. However, this small amount of data regarding the rupture time of cells does allow us to gain preliminary knowledge of λ and γ, since in our model the rupture rate is proportional to the size of the bacterial population, which in turn depends on the replication rate of the bacteria. Therefore, we use the distributions obtained for λ and γ as prior distributions in the subsequent fitting to the Kang et al. data. These distributions seem to agree well for the two different germination hypotheses and are shown in **Figure 2**. We also show that these preliminary estimates lead to a good representation of the rupture dynamics. Furthermore, the median of the distribution for λ is around 0.9 (*bacteria*·*h*)^-1^, which is consistent with the doubling time of 0.78 hours, measured by Kalns et al. (27) and used as an estimate in the within-host model by Day et al. (17). For more details on how the prior distributions in **Figure 2** were obtained, see the **Supplementary Material**. Uniform prior distributions are considered for the remaining parameters, as reported in **Table 3**. We note that these parameters are log-transformed because the prior range spans multiple orders of magnitude. For the model with two types of spores, we fixed *g_A_* > *g_B_* to represent that, without any loss of generality, type A spores are the ones with a faster germination rate. In order to sample parameter values *g_A_* and *g_B_* with priors reported in **Table 3**, and under the constraint *g_A_* > *g_B_*, we follow the ideas from (28).

**Table 3:** Prior distributions used in the ABC-SMC for the model with continuous heterogeneity of germination rate (top) and the model with two types of spores (bottom).

Parameter	Units	Description	Prior distribution
Model with continuous germination rate distribution			
μ *_g_*	*h* ^-1^	Mean of the normal distribution for *G*	$\log_{10}{\overset{\;}{\mu }}_{g}∼$ U(-2, 1)
σ*_g_*	*h* ^-1^	Standard deviation of the normal distribution for *G*	$\log_{10}{\overset{\;}{\sigma }}_{g}∼$ U(-2, 0.15)
$\tilde{\mu }$	*h* ^-1^	Death rate of newly germinated bacteria	$\log_{10}\tilde{\mu }∼$ U(-4, 1)
λ	(*bacteria*·*h*)^-1^	Replication rate of vegetative bacteria	See **Figure 2** (top)
μ	(*bacteria*·*h*)^-1^	Death rate of vegetative bacteria	$\log_{10}\overset{\;}{\mu }∼$ U(-4, 1)
γ	(*bacteria*·*h*)^-1^	Rupture rate	See **Figure 2** (top)
Model with discrete Bernoulli germination rate distribution			
*ε*	–	Probability that a given spore is of type A	*ε* ~ U(0, 1)
*g_A_*	*h* ^-1^	Germination and maturation rate of spores of type A	log_10_ *g_A_* ~ U(-4, 1)
*g_B_*	*h* ^-1^	Germination and maturation rate of spores of type B	log_10_ *g_B_* ~ U(-4, 1)
$\tilde{\mu }$	*h* ^-1^	Death rate of newly germinated bacteria	$\log_{10}\tilde{\mu }∼$ U(-4, 1)
λ	(*bacteria*·*h*)^-1^	Replication rate of vegetative bacteria	See **Figure 2** (bottom)
μ	(*bacteria*·*h*)^-1^	Death rate of vegetative bacteria	$\log_{10}\overset{\;}{\mu }∼$ U(-4, 1)
γ	(*bacteria*·*h*)^-1^	Rupture rate	See **Figure 2** (bottom)

**Figure 3:**
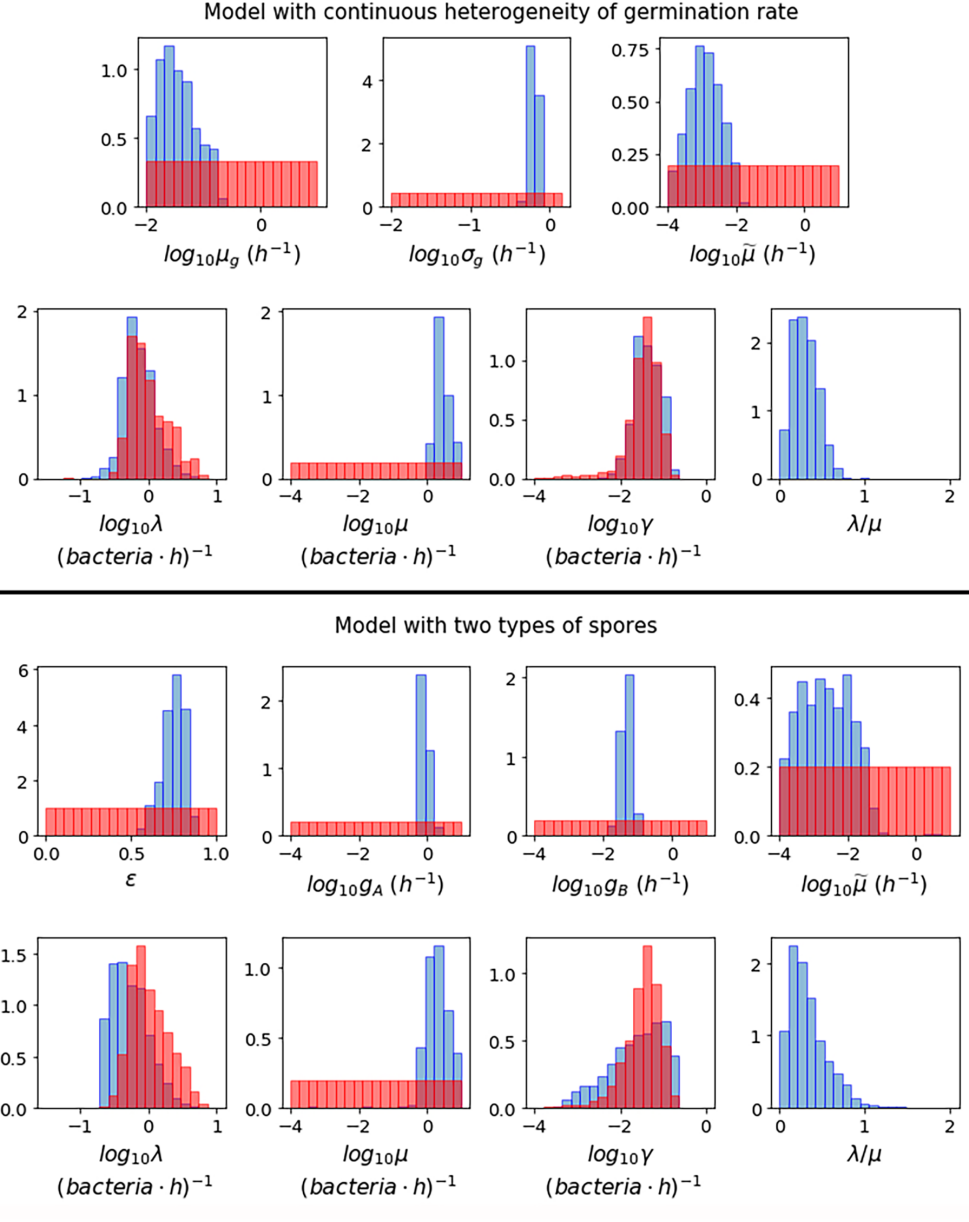
Prior distributions (red) and posterior histograms (blue) when performing ABC-SMC for the model with continuous heterogeneity of germination rate (top), and the model with two types of spores (bottom), using data from (23) of the number of intracellular spores and bacteria at different time points for MOIs 1:2, 1:10 and 1:20.

**Figure 3** shows the posterior histograms obtained by performing ABC-SMC for the two hypotheses considered, using the Kang et al. data, while summary statistics for these posteriors are reported in **Table 4**. By comparing the posterior histograms in blue with the prior distributions in red, we can see that it has been possible to learn significantly about most of the parameters for both hypotheses. For the model with continuous germination rate distribution, the value of *µ_g_*, corresponding to the most likely value for the germination rate of a given spore, is likely to be between 10^-2^ and 10^-1^ *h* ^-1^. For the model with two germination rates, the value of *ε* is likely to be between 0.5 and 1, so that the majority of the spores will germinate with rate *g_A_*, which is likely to be of the order of 10^-1^ *h* ^-1^, and the rest will germinate with rate *g_B_*, which is likely to be of the order of 10^-2^ *h* ^-1^. For both hypotheses we learn that the death rate of newly germinated bacteria, $\tilde{\mu }$, is likely to be very small. For the model with continuous heterogeneity in the germination rate, the posterior histograms for λ and γ show that the value of these parameters that produce a good match between this model and the Kang et al. data, are similar to the values that gave a good fit to the data from Akoachere et al. that we used to inform our priors for these parameters. For the model with two types of spores, the posterior histogram for λ is shifted slightly to the left from the prior distribution. For both hypotheses we have been able to learn significantly about the death rate of bacteria, *µ* and these accepted values are usually larger than the corresponding values for the replication rate, λ. This is shown in the posterior histograms for the ratio between the birth and death rate of bacteria, λ/*µ*, which mostly contain values less than 1, indicating that the bacteria are likely to die more quickly than they replicate.

**Table 4:** Summary statistics for the posterior sample of each parameter, shown in blue in **Figure 3**, for the model with continuous heterogeneity of germination rate (top) and two types of spores (bottom).

Parameter	Units	Min	Median	Mean	Max	95% Credible Interval
Model with continuous germination rate distribution						
*µ_g_*	*h* ^-1^	1.00 × 10^-2^	3.30 × 10^-2^	3.60 × 10^-2^	2.38 × 10^-1^	(1.16 × 10^-2^, 1.60 × 10^-1^)
σ*_g_*	*h* ^-1^	4.36 × 10^-1^	6.27 × 10^-1^	6.23 × 10^-1^	8.43 × 10^-1^	(5.01 × 10^-1^, 7.65 × 10^-1^)
$\tilde{\mu }$	*h* ^-1^	1.01 × 10^-4^	1.18 × 10^-3^	1.20 × 10^-3^	1.73 × 10^-2^	(1.50 × 10^-4^, 9.58 × 10^-3^)
λ	(*bacteria*·*h*)^-1^	1.35 × 10^-1^	6.93 × 10^-1^	7.28 × 10^-1^	4.96 × 10^0^	(2.76 × 10^-1^, 2.43 × 10^0^)
*µ*	(*bacteria*·*h*)^-1^	9.70 × 10^-1^	2.55 × 10^0^	2.81 × 10^0^	9.66 × 10^0^	(1.31 × 10^0^, 7.49 × 10^0^)
γ	(*bacteria*·*h*)^-1^	3.60 × 10^-3^	3.94 × 10^-2^	4.15 × 10^-2^	1.75 × 10^-1^	(1.07 × 10^-2^, 1.33 × 10^-1^)
Model with discrete Bernoulli germination rate distribution						
*ε*	–	5.29 × 10^-1^	7.54 × 10^-1^	7.45 × 10^-1^	9.06 × 10^-1^	(5.93 × 10^-1^, 8.48 × 10^-1^)
*g_A_*	*h* ^-1^	4.86 × 10^-1^	8.16 × 10^-1^	8.50 × 10^-1^	2.74 × 10^0^	(5.37 × 10^-1^, 1.74 × 10^0^)
*g_B_*	*h* ^-1^	1.18 × 10^-3^	4.90 × 10^-2^	4.70 × 10^-2^	1.04 × 10^-1^	(2.21 × 10^-2^, 8.76 × 10^-2^)
$\tilde{\mu }$	*h* ^-1^	1.02 × 10^-4^	2.16 × 10^-3^	2.27 × 10^-3^	5.03 × 10^0^	(1.27 × 10^-4^, 4.25 × 10^-2^)
λ	(*bacteria*·*h*)^-1^	2.01 × 10^-1^	4.94 × 10^-1^	5.34 × 10^-1^	4.28 × 10^0^	(2.14 × 10^-1^, 1.95 × 10^0^)
*µ*	(*bacteria*·*h*)^-1^	4.97 × 10^-4^	1.93 × 10^0^	2.00 × 10^0^	9.98 × 10^0^	(5.82 × 10^-1^, 8.16 × 10^0^)
γ	(*bacteria*·*h*)^-1^	4.13 × 10^-4^	2.97 × 10^-2^	2.34 × 10^-2^	2.24 × 10^-1^	(9.63 × 10^-4^, 1.89 × 10^-1^)

**Figure 4:**
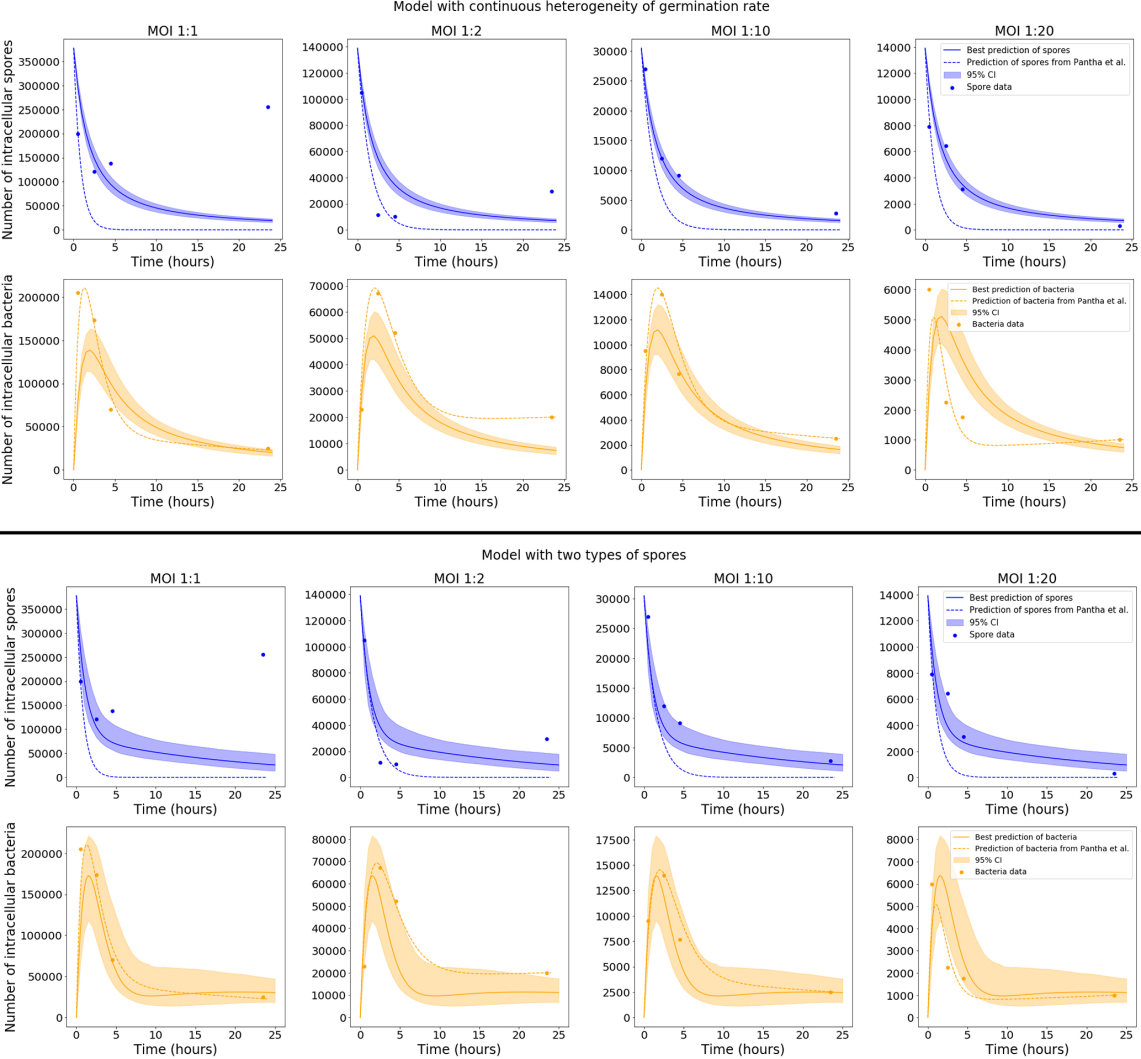
Plots showing the best predictions (solid lines) and pointwise 95% credible intervals (shaded regions) of the time-courses for the mean number of intracellular spores and bacteria, for the model with continuous heterogeneity of germination rate (top), and the model with two types of spores (bottom). The best predictions are the model outputs obtained by using the accepted parameter set with the smallest distance to the data, and the pointwise 95% credible intervals show the uncertainty in those predictions, given the range of parameter values in the 10^3^ accepted parameter sets from the final iteration of ABC-SMC. The predictions from the model by Pantha et al., using separate sets of parameter estimates for each MOI, are shown as dashed lines. For our model, only the data for MOI 1:2, 1:10 and 1:20 were used in the ABC-SMC to calibrate model parameters. The comparison of the model predictions with the MOI 1:1 data is shown here as a qualitative model validation.

Model predictions were obtained for each accepted parameter set from the ABC-SMC. **Figure 4** shows the pointwise 95% credible intervals of these time-courses, which indicate the uncertainty in the mean number of intracellular spores and bacteria from the model, due to the range of accepted parameter sets. The solid lines show the model output for the parameter sets with the smallest distance to the data, referred to as the best model predictions. For the model with continuous germination rate distribution, the predictions are close to the data at some time points, but overall do not seem to explain the data very well, since the peak of intracellular bacteria in the model predictions seems to be lower than the peak indicated by the data. On the other hand, the predictions of the model with two types of spores show a fairly good agreement with all data sets. It seems that this latter model, with two discrete germination rates, is better able to describe the pattern of biphasic decay in the number of spores seen in the data. This model also explains the bacterial data significantly better than the hypothesis of continuous germination rate distribution.

Only the data for MOI 1:2, 1:10 and 1:20 were used in the ABC-SMC analysis to calibrate model parameters. Still, we compare our calibrated model predictions with the MOI 1:1 data, by making use of the corresponding initial condition for this MOI, *S*_0_ = 377500. For the model with two types of spores, the best prediction is very close to the MOI 1:1 data at some time points, which demonstrates a possibility that the model could be used to extrapolate between MOIs. However, the assumption that each cell will only phagocytose a single spore is less viable at MOI 1:1, so the model may need to be adapted slightly in order to explain the dynamics at higher MOIs. We note that the long-term behaviour of spores for MOI 1:1, where a significant unexpected increase in the number of spores over time is observed, was not explained by Kang et al. (23) or Pantha et al. (15), and cannot be mechanistically explained by our intracellular infection model either, regardless of the choice of parameters.

**Figure 4** also shows, as dashed lines, the predictions of the model by Pantha et al. using their parameter estimates. When we compare these to our predictions, it seems that with the consideration of heterogeneity in the germination rate, our model provides a better explanation for the number of intracellular spores seen in the data, especially for MOI 1:10 and MOI 1:20. However, the fit of our model to the bacterial data does not look quite as good as the predictions by Pantha et al. This is not surprising, since Pantha et al. allow different parameter values for each data set, meaning that their model has more parameters and higher complexity, giving it more freedom to fit the data. To compare the goodness-of-fit of the models, we use the Corrected Akaike’s Information Criterion (AIC*_C_*), which penalises models with a higher number of parameters if there is not enough improvement in the goodness-of-fit to warrant the additional complexity. Roughly speaking, lower values of the AIC*_C_* indicate a better fit to the observed data. We have calculated the value of AIC*_C_* for the model by Pantha et al. and the two versions of our model with different distributions for the germination rate. For details about how the AIC*_C_* was calculated see the **Supplementary Material**. For our models the value of AIC*_C_* was calculated using the parameter set that gave the smallest distance in the ABC-SMC, obtaining a value of AIC*_C_* = 1.42 for the model in which the germination rate is a continuous random variable, and a value of AIC*_C_* = -3.8 for the one with two types of spores. For the model by Pantha et al., the value of AIC*_C_* was calculated using the parameter estimates reported in (15, Table 7), giving a value of AIC*_C_* = 176.73. Our models have a lower AIC*_C_* than the model by Pantha et al., mainly because we used the same parameter estimates for each MOI, whereas Pantha et al. obtained separate estimates for each MOI, meaning that they have many more free parameters. Even with the inclusion of heterogeneity in the germination rate, the version of our model with continuously distributed germination rates is not able to properly capture all the data. Instead, a bi-modal model of heterogeneity in the germination rate is needed to explain both the spore and the bacterial data. This is supported by the fact that the AIC*_C_* value is lower for the discrete Bernoulli hypothesis, even though it has one more parameter. This indicates that this model may explain the data better than the model with continuously distributed germination rates.

## Results

The results of the parameter calibration suggest that the hypothesis of germination rate heterogeneity with two discrete types of spores is better supported by the data than the model with continuous heterogeneity. The model with two types of spores is able to describe the biphasic decay seen in the spore data, especially for MOIs 1:10 and 1:20, as well as the behaviour of the bacterial data. Therefore, in this section we will only focus on the model with two types of spores. The set of parameter values that gave the smallest distance to the data in the ABC-SMC is provided in **Table 5**. We have used these parameter values to calculate the various descriptors of the model discussed in the *Materials and Methods* section, and have also investigated the effect of the uncertainty in the parameter values indicated by the posterior distributions obtained from ABC-SMC.

**Table 5:** Parameter values that gave the smallest distance between the two types of spores model and the data from (23) in the ABC-SMC analysis.

Parameter	Units	Description	Value
ε	–	Probability that a given spore is of type A	0.778846
*g_A_*	*h* ^-1^	Germination and maturation rate of spores of type A	0.894274
*g_B_*	*h* ^-1^	Germination and maturation rate of spores of type B	0.046794
$\tilde{\mu }$	*h* ^-1^	Death rate of newly germinated bacteria	0.003502
λ	(*bacteria*·*h*)^-1^	Replication rate of vegetative bacteria	0.643111
*μ*	(*bacteria*·*h*)^-1^	Death rate of vegetative bacteria	1.637989
γ	(*bacteria*·*h*)^-1^	Rupture rate	0.043792

**Figure 5:**
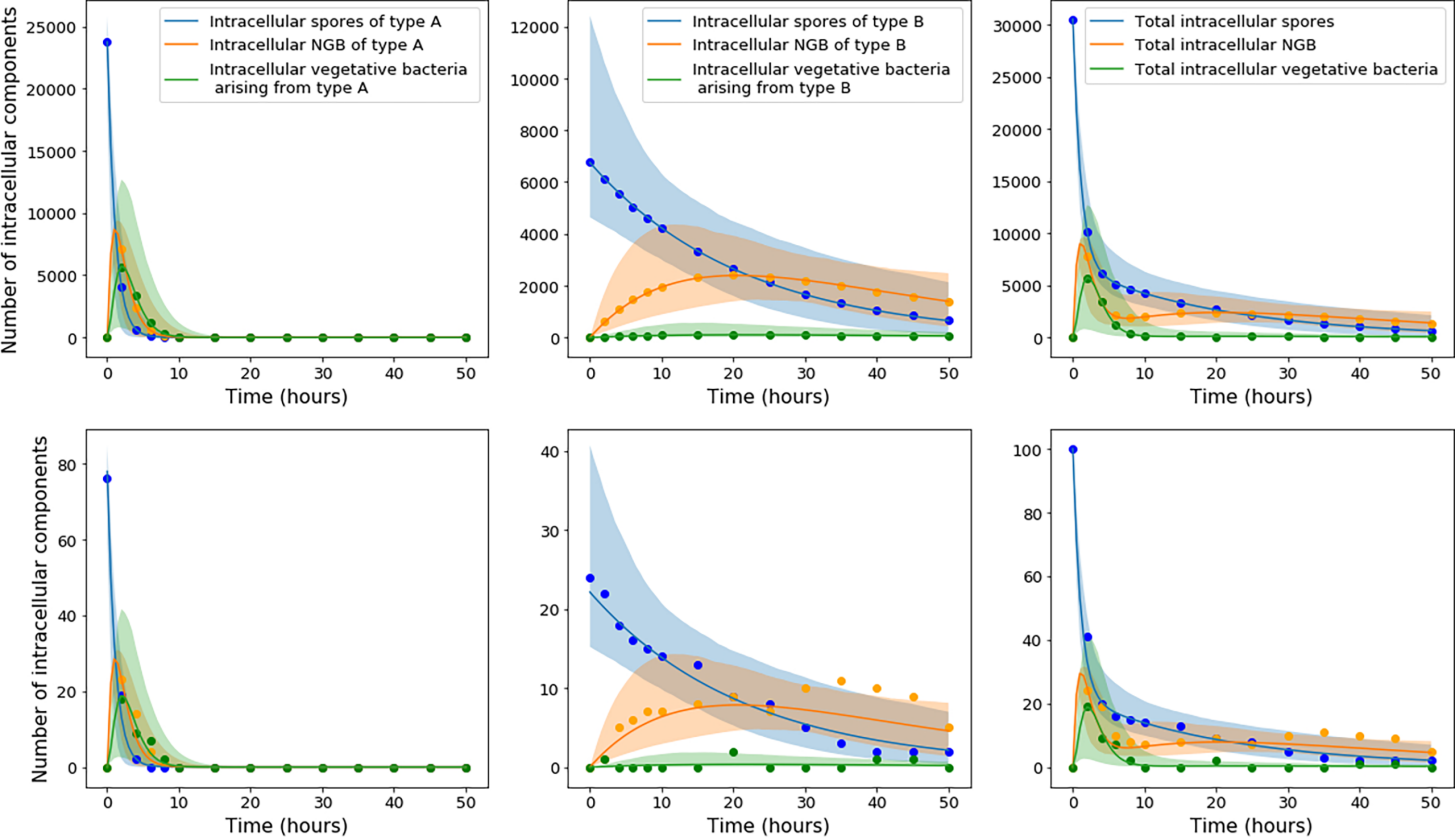
The top row of plots corresponds to a population of *S*_0_ = 30500 infected cells, each containing a single spore at time 0, whereas the bottom row corresponds to an initial condition of *S*_0_ = 100. Left: The mean number of type A spores, ε*S*_0_ *p*_1_*S*__(*t*; *g* = *g_A_*) , type A newly germinated bacteria, ε*S*_0_ *p*_1_*NGB*__(*t*; *g* = *g_A_*), and vegetative bacteria, ε*S*_0_ *B_V_*(*t*; *g_A_*) = ε*S*_0_ $\sum_{i=1}^{\infty }$ *i p_i_*(*t*; *g* = *g_A_*), arising from the type A spores in the infected macrophages. Centre: The analogous functions for the populations arising from the initial spores with germination rate *g_B_*. Right: The overall mean number of spores, *S*_0_ *S*(*t*), newly germinated bacteria, *S*_0_ *B_NGB_*(*t*), and vegetative bacteria, *S*_0_ *B*_ν_(*t*), obtained by adding together the populations for each type of spore. The solid lines indicate the means for the estimated parameter values in **Table 5**, while the shaded regions indicate the pointwise 95% credible intervals for these means, when the uncertainty in the parameter values from the posterior distributions is taken into account. The equations used to compute these curves were Eqs. (5), (6), (11), (12), (41), and (42). The dots show values for the size of the different populations over time from a single stochastic simulation beginning with *S*_0_ spores.

In the *Spore Germination* section, we found expressions for the probabilities, $p_{1_{S}}(t)\text{ and }p_{1_{NGB}}(t)$, that an infected macrophage will contain a spore or newly germinated bacterium, respectively, at time *t* ≥ 0, given that the macrophage contained a spore with germination rate *g* at time 0. Note that since in our model the macrophage is assumed to only contain at most one spore or newly germinated bacterium at any one time, these probabilities are equal to the mean number of spores and newly germinated bacteria inside the macrophage at time *t* ≥ 0. In the *Cellular Fate: Rupture or Recovery* section we also explained how to calculate the mean number of vegetative bacteria, *B*_ν_(*t*; *g*), in an infected macrophage at time *t* ≥ 0, given that the macrophage contained a spore with germination rate *g* at time 0. One can then consider a population of *S*_0_ independent infected cells, each containing a single spore at time 0. Assuming that each initial spore can have one of two possible germination rates (either rate *g_A_* with probability *ε*, or rate *g_B_* with rate 1 - *ε*), one can calculate a time course for the total mean number of intracellular spores, newly germinated bacteria, and vegetative bacteria, for a population of infected cells, split into the populations arising from each of the two types of spores. This is depicted in **Figure 5**, where the first column corresponds to the populations arising from spores of type A, the second column corresponds to the populations arising from spores of type B, and the third column shows the sum of the populations from both types of spores. The solid lines indicate the means for the estimated parameter values in **Table 5**, while the shaded regions indicate the pointwise 95% credible intervals for these means, when the uncertainty in the parameter values from the posterior is considered. The first two plots show two very different timescales for the dynamics of each kind of spore, and when these populations are added together in the third plot, one can observe the biphasic behaviour in the number of spores that is observed in the data from (23). The blue curve here indicates the prediction from the model for the total mean number of intracellular spores over time. The other two curves indicate the predictions for the mean number of intracellular newly germinated bacteria (orange), and vegetative bacteria (green), so that when these are added together, one obtains the prediction for the total number of intracellular bacteria, as shown in the predictions from the parameter calibration in **Figure 4**. The top row of plots in **Figure 5** corresponds to an initial condition of *S*_0_ = 30500 spores, equal to the initial condition from the MOI 1:10 data used in the ABC-SMC. On the other hand, the bottom row corresponds to an initial condition of *S*_0_ = 100. In both cases, the coloured dots indicate the sizes of the different populations over time, from a stochastic simulation of the model starting with *S*_0_ spores. The results from the simulations show that when there are many initial spores (top row), the behaviour is very deterministic, but when the number of initial spores is relatively small (bottom row), there is much more stochasticity. This stochasticity could be relevant in *in vivo* settings, where infection might depend on a small group of spores germinating and producing a relatively small number of bacteria, as discussed in the *Discussion* section.

**Figure 6:**
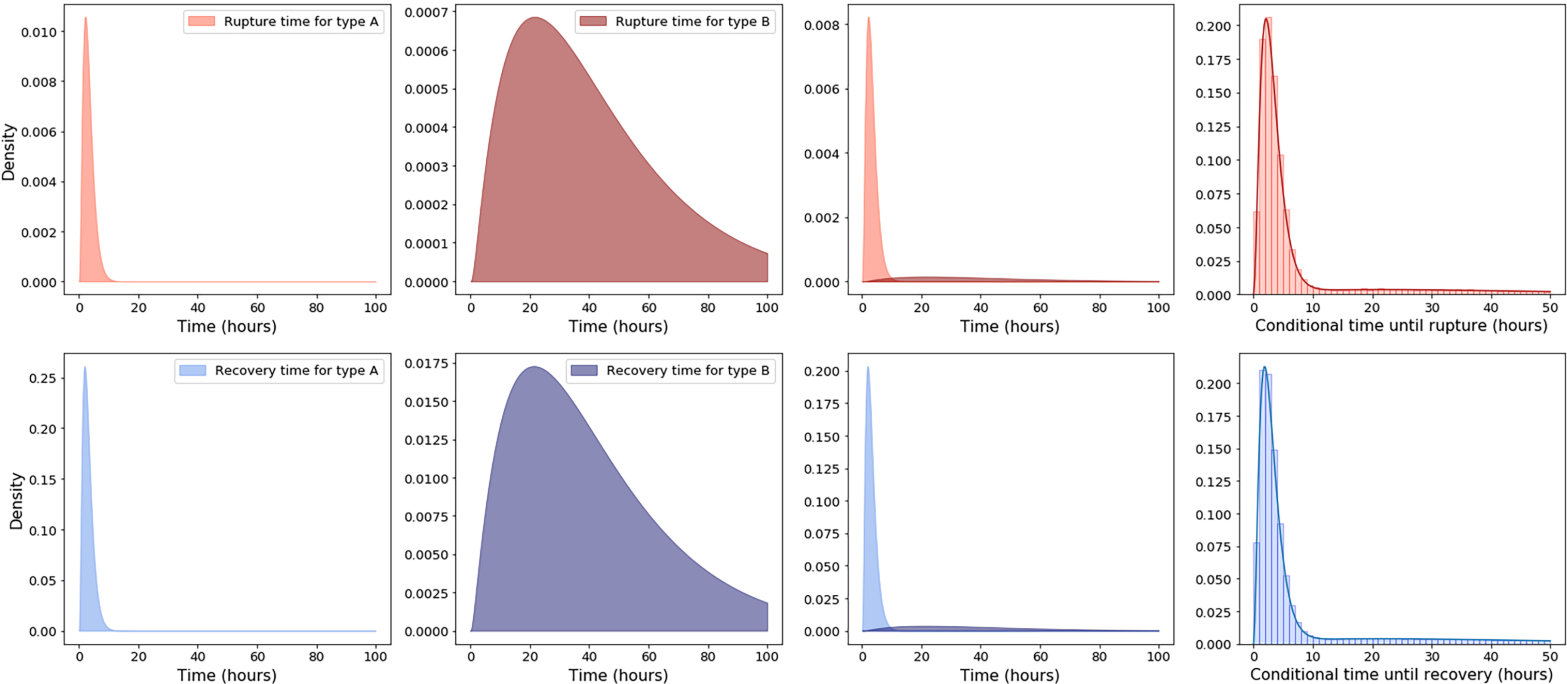
Top row, from left to right, the first two plots show the probability density functions for the rupture time of a macrophage infected with a spore of type A and type B, respectively, given by *f*_$T_{1_{S}}^{R}$_ (*t*; *g_A_*) and *f*_$T_{1_{S}}^{R}$_ (*t*; *g_B_*). The third plot shows these densities on the same plot, when they are scaled by the relative frequencies of each germination rate: *εf*_$T_{1_{S}}^{R}$_ (*t*; *g_A_*) and (1 − *ε*)*f*_$T_{1_{S}}^{R}$_ (*t*; *g_B_*). The fourth plot shows as a solid line the probability density function for the rupture time of a macrophage infected with a single spore, conditioned on rupture occurring, which is given by *f*_$T_{1_{S}}^{R}$_ (*t*)/$r_{1_{S}}^{R}$. Also shown on the fourth plot is a histogram of the finite rupture times from 10^6^ stochastic simulations of the model in **Figure 1**. Plots on the bottom row correspond to the analogous densities for the time to recovery of an infected macrophage. The estimated parameter values in **Table 5** were used to compute these functions.

**Figure 7:**
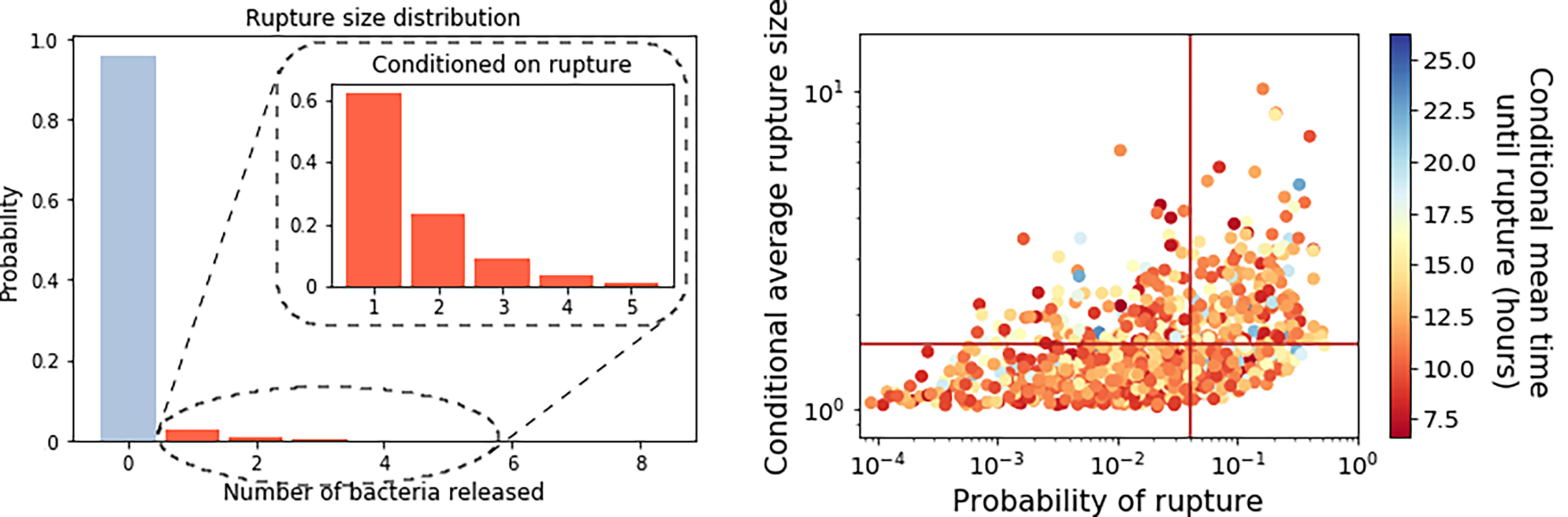
Left: The best predicted rupture size distribution for the model with two types of spores, computed using Eq. (49), with the estimated parameter values from **Table 5**. Inset is the conditional rupture size distribution, for the number of bacteria released by a macrophage infected with a single spore, given that it ruptures rather than recovers. Right: Scatter plot of the probability of rupture against the expected rupture size (conditioned on rupture occurring), for each parameter set in the posterior distribution. Lines indicate the values that correspond to the parameter set from **Table 5**. The colour of the points indicates the conditional mean time to rupture for each parameter set considered.

In the *Cellular Fate: Rupture or Recovery* section we explained how to calculate the probability density functions for the times to recovery and rupture of a macrophage initially infected with one spore. The probability density functions for the time to rupture are plotted along the top row of **Figure 6**, for the inferred parameter values in **Table 5**. From left to right, the first two plots show the density functions for the time to rupture of an infected cell containing a spore of type A and type B, respectively. We observe very different rupture timescales for each kind of spore. The third plot shows these densities on the same plot, when they are scaled by the relative frequencies of each germination rate, so that the sum of these two densities gives the overall probability density function for the rupture time of a cell infected with a single spore. Note that this probability density function does not integrate to 1, but instead the probability of rupture. Nevertheless, one can divide the density by the probability of rupture, giving the conditional density function of rupture time, shown as a solid line in the fourth plot. Also shown on the fourth plot is a histogram of the finite rupture times from 10^6^ stochastic simulations of the model in **Figure 1**. On the bottom row of **Figure 6** are the analogous functions for the recovery time. Interestingly, the conditional probability density functions for the rupture and recovery times are almost identical. This is likely due to the fact that these timescales are heavily dominated by the germination time of the spore, and once the spore has germinated, rupture or recovery of the phagocyte happens relatively quickly. One can also compute the conditional mean times to rupture or recovery of a macrophage infected with a single spore, which are the means of the rightmost histograms in **Figure 6**. For the parameter values in **Table 5**, the conditional mean rupture and recovery times are approximately 11.3 hours and 11.6 hours, respectively. However, the uncertainty in the parameter values, shown in the posterior distributions, leads to uncertainty in these timescales. For instance, this is indicated by the range of conditional mean times until rupture shown in the plot on the right of **Figure 7**.

The probability distribution for the number of bacteria released by an infected macrophage, for the parameter values in **Table 5**, is provided in **Figure 7** (left). The probability that no bacteria are released by the macrophage is predicted to be 0.96, which suggests that most macrophages will be able to recover and eliminate the intracellular infection, and that we would expect only 4% of infected macrophages to eventually rupture and release bacteria. The results also indicate that when macrophages do rupture, they will only release a few bacteria, with an average of 1.6 bacteria released from a macrophage that ruptures. This is consistent with the fact that a high dose of spores is required for infection, reported to be between 8 × 10^3^ and 5 × 10^4^ spores (29). Cote et al. (30) explain that although macrophages kill most of the germinated bacteria that they encounter, a low percentage of bacteria survive the antimicrobial environment in the macrophage and escape to begin the extracellular infection. This is further supported by findings from Jones et al. (31), who observed that after infection of a guinea pig with 10^7^ spores, 99% of the germinated spores were killed within an hour, but the 1% that survived managed to replicate extracellularly and ultimately reached a concentration of 10^8^ bacteria/ml in the blood at the time of death.

**Table 5** reports our best prediction for the parameter values according to the distance in the ABC-SMC. Yet, the advantage of a Bayesian approach in the parameter calibration is that it quantifies the uncertainty in the parameter values, which translates into the uncertainty in the descriptors of the model. The scatter plot on the right of **Figure 7** shows the probability of rupture plotted against the average of the conditional rupture size distribution, for each parameter set in the posterior sample shown in **Figure 3** (bottom), with the lines indicating the corresponding values for the parameter set in **Table 5**. There is a positive correlation between these two descriptors, indicating that if macrophages are more likely to rupture, they are also likely to release more bacteria when they do rupture. Parameter sets leading to a very small probability of rupture will likely correspond to death rates much larger than the replication rate. Conversely, parameter sets leading to a larger probability of rupture correspond to bacterial death rates closer to the replication rate. This would allow for greater bacterial replication on average before rupture, and in turn a larger average rupture size. The colour of the points on this scatter plot indicates the conditional mean time until rupture for each parameter set. This illustrates the uncertainty of the timescale for rupture, with the possible mean rupture times from the posterior distributions ranging from around 7.5 to 25 hours. Furthermore, it is possible to find pairs of parameter sets that give differing average rupture times, but with similar probabilities of rupture and conditional average rupture sizes.

## Discussion

We propose a stochastic model for the dynamics of *B. anthracis* spores and bacteria inside an infected phagocyte. One of the main features of our model is the consideration of heterogeneity in the germination rate of spores. Two hypotheses were considered to characterise this heterogeneity. The first hypothesis was that the germination rate is continuously distributed in a population of spores and follows a truncated normal distribution. The second hypothesis was that the spore population can be split into two kinds that germinate at different rates. We carried out parameter calibration, for each hypothesis, by means of Approximate Bayesian Computation Sequential Monte Carlo (ABC-SMC) (22). Our results suggest that the discrete germination hypothesis is better supported by the data, since the model with this distribution of germination rates allows us to account for the biphasic decline seen in the spore counts, as well as the observed behaviour of the bacterial counts. This assumption of two types of spores also agrees with experimental evidence showing that in some *Bacillus* spore populations, a subset of the spores germinate much more slowly than the average spore, and are termed superdormant (20). This leads to qualitatively different predictions for the mean number of spores over time in a population of *in vitro* cells, compared to previous theoretical predictions made in (15), as shown in **Figure 4**. Although our posterior estimated values of the germination rate of spores of type A are similar to the germination rates predicted in (15), our model predicts that a subset of spores will germinate much more slowly than this. We note that the “toy” discrete distribution with two rates, *g_A_* and *g_B_*, considered here, is a first step and more complex descriptions of the germination rate heterogeneity will be considered in future work. Experimental quantification of germination times would allow us to explore and potentially validate these more complex models. A limitation of our model is that the same rate is considered for each step of the germination-maturation process, compared to the model by Pantha et al., where a separate rate, *m*, was considered for the maturation step. However, Pantha et al. did not calibrate this rate *m*, and instead performed a sensitivity analysis. Since we are limited in the available experimental data, it would be difficult to calibrate different rates for each germination-maturation step. However, if further data were to become available which allowed one to distinguish between newly germinated and vegetative bacteria, then a separate rate could be incorporated for the maturation step of the germination process. This would also allow for more complicated distributions to account for heterogeneity in the germination and maturation rates.

Another important feature of our model is the consideration of rupture of infected phagocytes. This means that different behaviours can be described by our model compared to the model by Pantha et al., since in our model there is a chance that some intracellular bacteria will survive the microbicidal environment of the phagocyte and cause the cell to rupture. In the experiment by Kang et al., if bacteria were released into the extracellular medium then they would have been washed away before intracellular numbers of spores and bacteria were measured, and hence the decrease of intracellular bacteria seen in the data may not have been purely due to macrophage-induced killing of bacteria but may have been due to the release of intracellular bacteria from dying cells. Further data including information about macrophage rupture versus survival would be needed in order to disentangle these processes. Our stochastic model (see **Figure 1**) has allowed us to compute the probability that an infected cell will eliminate the infection and recover, and the probability that an infected cell will rupture and release its bacterial content. We have also computed the mean time for an infected cell to reach one of these two fates, conditioned on the event occurring. The probability distribution for the number of bacteria released by an infected macrophage has also been calculated. By calibrating the parameters using *in vitro* experimental data, the rupture size distribution shown in **Figure 7** is able to capture the fact that the majority of spores taken up by macrophages are likely to be eliminated by the host cell, releasing no bacteria, but a few germinated spores may survive phagocytosis, leading to death of the host cell and release of a small number of bacteria. This is in agreement with recent experimental work (13). Although we have parametrised our model with data from a study that used macrophages, there is also evidence to suggest that dendritic cells play a role in the early infection stages of anthrax (10). Therefore, it will be important to also consider this cell type in future. If *in vitro* infection data for dendritic cells becomes available, it would be relevant to re-parametrise the model with such data to investigate the differences between the roles of the two host cell types in anthrax disease. For example (32) indicates that dendritic cells may not be as capable as macrophages in their abilities to reduce bacterial numbers.

**Figure 8:**
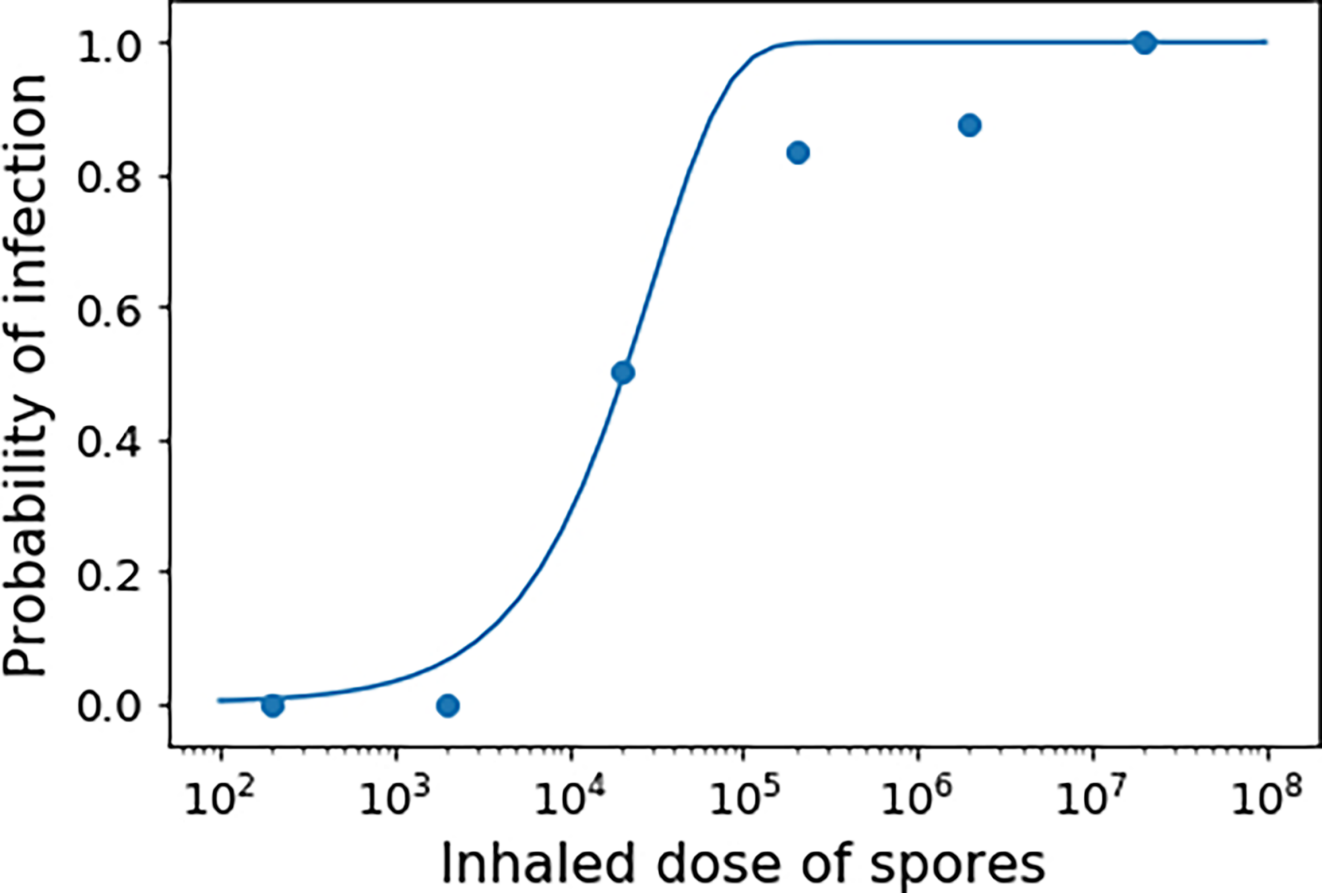
Exponential dose-response curve fit to the Altboum et al. guinea-pig Vollum strain dose-response data set (33). Here the probability that one inhaled spore will cause a response is set to *r* = 3.31 × 10^-5^.

The intracellular model presented here could be used in mechanistic within-host models of anthrax infection to describe the dynamics of *B. anthracis* within the lung and lymph nodes of an individual, following inhalation of some initial dose of spores, such as the one by Day et al. in (17). The stochastic nature of the intracellular model presented here could allow such within-host models to consider inter-phagocyte variability in rupture size by incorporating the rupture size distribution into the within-host infection dynamics. Heterogeneity of the rupture size has been shown to be important in a similar model for the pathogen *F. tularensis* (25). Furthermore, a within-host model could be linked to dose-response data and used to investigate the individual infection risk given an initial inhalational dose. A standard approach in dose-response assessment is the use of single-hit models. These models assume that when an individual is infected with a pathogen, the organisms act independently in the host so that the probability that any one organism in the initial dose produces an eventual infection is independent of the size of the dose, and the probability of infection is equivalent to the probability that at least one of the organisms in the initial dose will lead to an infection. For example, in the simple exponential model, for an average initial dose *D*, the probability of infection is given by, *I*(*D*; *r*) = 1 – *e* ^-^ *^rD^*, where *r* is the probability that a single organism will produce a response. This exponential model can be fitted to data in order to estimate *r*. **Figure 8** shows the exponential dose-response curve compared to the Altboum et al. guinea-pig Vollum strain dose-response data set (33). For this data set the value of *r* that gives the best fit is around *r* = 3.31 × 10^-5^. However, the specific value of *r* obtained varies greatly, depending on the data set used to calibrate the dose-response curve. In particular, different anthrax dose-response data sets lead to a range for the ID_50_ (the dose of spores such that the probability of infection is equal to 0.5) of about 10^3^ to 10^5^. These differences could be due to changes in susceptibility for each animal species used or in virulence for each anthrax strain used (34).

Dose-response models for anthrax already exist, such as the competing risks model developed by Brookmeyer et al. (35). This is an example of a single-hit model in which *r* is taken to be, $r=\frac{\lambda }{\lambda +\theta }$, where λ is the germination rate of spores and *θ* the rate at which spores are cleared from the lungs. The hypothesis of the competing risks model for anthrax is that if a single spore survives ingestion by a macrophage and successfully germinates without getting cleared, then the resulting bacterium will be certain to cause an infection. However, there is evidence that neutrophils can kill vegetative anthrax bacteria (36), which means that once a spore in an infected cell has germinated and a bacterium is released, there is no guarantee that the bacterium will survive and cause infection. Another simplification used in the competing risks model is that germination and clearance of spores are both assumed to be exponential processes. However, this might not be the case. For instance, in our intracellular model, as well as the one by Pantha et al., the consideration of the newly germinated bacterium means that the total germination-maturation time is non-exponential.

**Figure 9:**
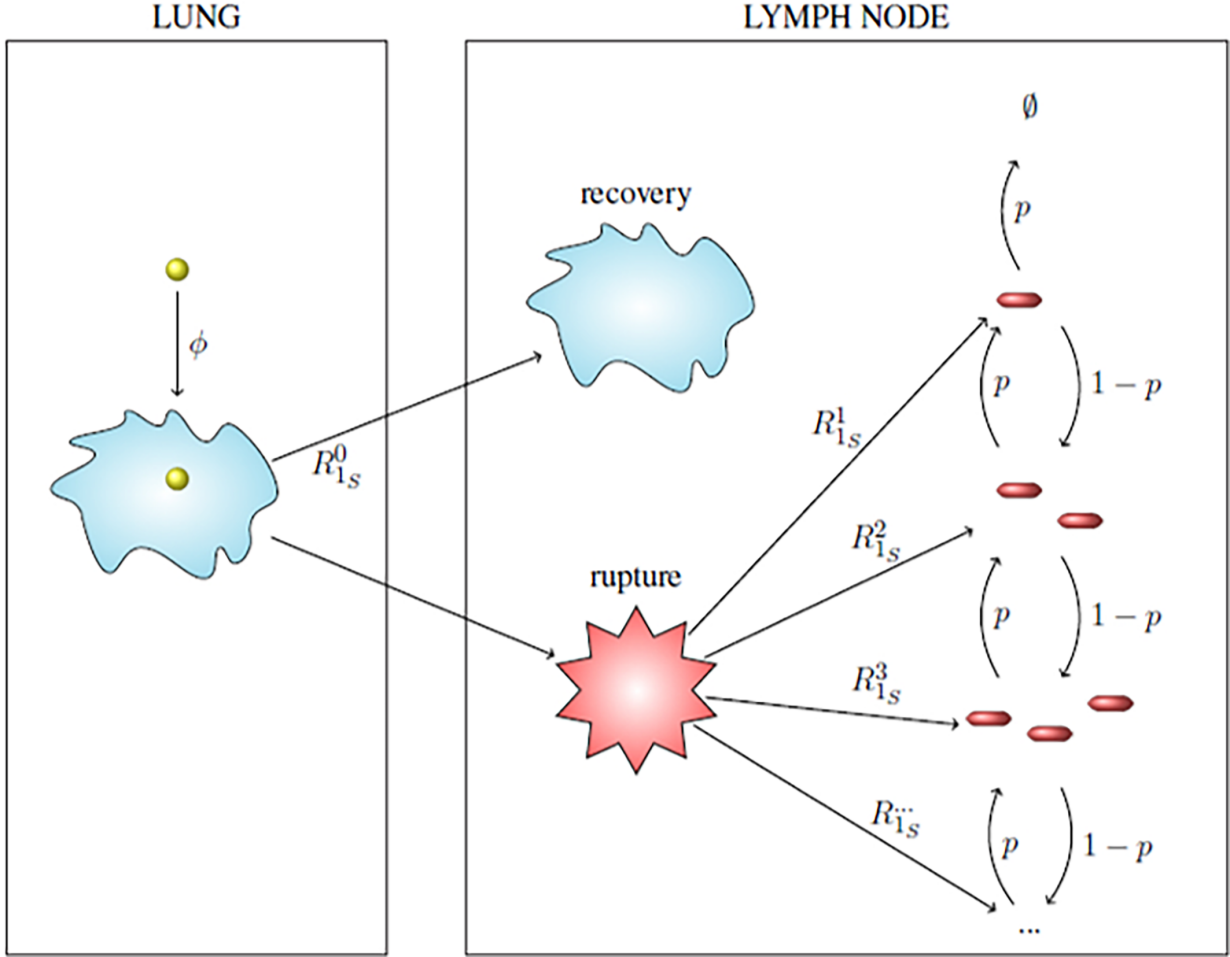
Toy model for the possible fates of a single spore in the early stages of inhalational anthrax infection. Within the lung, an inhaled spore (yellow ball) becomes deposited with probability *ϕ* and is ingested by a host phagocyte. The intracellular spore germinates and the phagocyte might kill the germinated bacterium or the bacterium may survive the antimicrobial environment, replicate and cause the phagocyte to rupture, as described in the intracellular model. The infected phagocyte migrates to the lymph nodes and either recovers or ruptures and releases some bacteria, according to the probabilities calculated with the intracellular model, $R_{1_{S}}^{n}$. The population of bacteria released follows a birth and death process, where an extracellular bacterium may be killed by host immune cells with probability *p* or a bacterium will replicate extracellularly with probability 1 - *p*, until either there are no extracellular bacteria remaining, or the population of extracellular bacteria reaches a threshold *M*.

The competing risks model involves parameters for the two competing processes of spore germination and spore clearance but does not explicitly consider macrophage rupture or intracellular bacterial dynamics. Hence, a fully mechanistic model, such as the intracellular model proposed here, could allow one to construct more detailed dose-response approaches which go beyond the simple competing risks assumptions, and explore the stochasticity of the rupture events, and the possibility that even if a few bacteria are generated, infection might not occur if these few bacteria are killed. **Figure 9** shows a “toy” representation of the possible fates of each spore in the early stages of inhalational anthrax infection in the lungs and lymph nodes. After inhalation, these spores must be transported through the respiratory system in order to reach the alveoli of the lungs, where they have the chance to cause an infection. Hence the initial dose of spores that is delivered to this area of the lungs may be much smaller than the original inhaled dose (37). Since the delivered dose may be very small compared to the exposed dose, the stochastic nature of the intracellular dynamics illustrated in **Figure 5** can become important. For the model by Brookmeyer et al. (35), the transport dynamics of spores through the respiratory system would be included in the clearance rate, *θ*. Alternatively, to explicitly account for the fact that some of the inhaled spores will not be delivered to the alveolated region of the lung, and to differentiate this from the clearance of spores by macrophages, one can assume that each inhaled spore has some probability, *ϕ*, of being deposited in the alveoli (18).

We assume that a spore deposited in the alveoli will be ingested by a host phagocyte, which will then migrate to the lymph nodes and either recover or rupture, releasing *n* ∈ ℕ bacteria with probability $R_{1_{S}}^{n}$ calculated from the intracellular model. When bacteria are released, each extracellular bacterium can be killed by host immune cells with probability *p* < 1, or proliferate extracellularly with probability 1 - *p*. Each inhaled spore has two possible fates: the response state and the clearance state. We assume that a spore will reach the response state if it manages to lead to a population of *M* ∈ ℕ bacteria in the lymph node. On the other hand, the infection from an inhaled spore can be cleared in one of three ways: if the spore is not deposited in the alveolated region of the lungs, if the spore is phagocytosed but the infected cell recovers rather than ruptures, or if the infected cell ruptures but the population of bacteria released from the cell becomes extinct before reaching the threshold *M*. From these assumptions, *r* is the probability that the fate of a single inhaled spore is the response state rather than the clearance state, and we can construct a formula for *r* that takes into account all of the mechanisms in the intracellular model, and the possibility that the few bacteria released from a rupturing cell may be killed by the host immune defences before they are able to proliferate to a sufficient number to cause a response. In this case, the probability that infection is established by a single inhaled organism can be given by

$$r={\{}_{\phi (\sum_{n=1}^{M-1}R_{1_{S}}^{n}\frac{n}{M}+\sum_{n=M}^{+\infty }R_{1_{S}}^{n}),\text{if }p=0.5.}^{\phi (\sum_{n=1}^{M-1}R_{1_{S}}^{n}\frac{1-{\left(\frac{p}{1-p}\right)}^{n}}{1-{\left(\frac{p}{1-p}\right)}^{M}}+\sum_{n=M}^{+\infty }R_{1_{S}}^{n}),\text{if }p\neq 0.5,}$$

**Figure 10:**
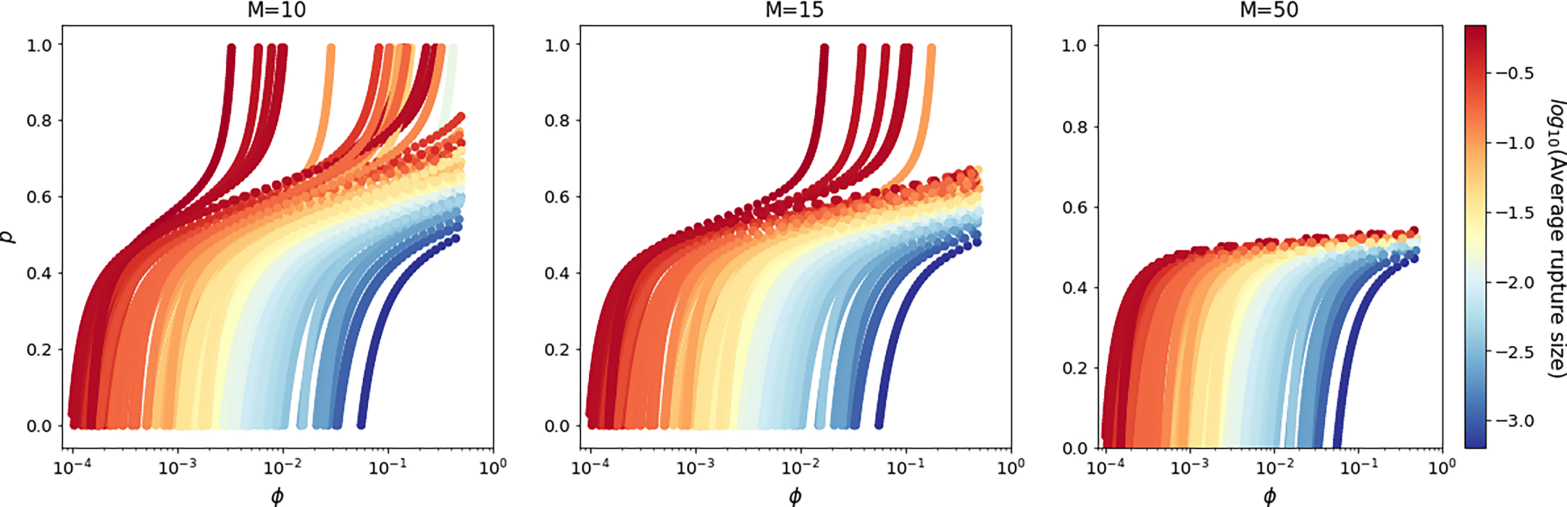
Combination of parameter values for *p* and *ϕ* that give *r* = 3.31 × 10^-5^, for three different values of the threshold number of bacteria, *M* = 10, 15, 50. The combinations are shown for different rupture size distributions, sampled from the posterior distribution of the intracellular model. The average of each rupture size distribution is indicated by the colour of the points.

The competing risks hypothesis is equivalent to taking *M* = 1. A value of *M* greater than one allows some bacteria to die extracellularly in the early stages of infection. We note that the parameter calibration in our intracellular model incorporates parameter uncertainty, encoded in the posterior distributions (see **Figure 3**). Thus, different parameter sets will lead to different predicted rupture size distributions. Furthermore, the deposition probability, *ϕ*, depends on a number of different factors, such as breathing rate, which will vary between individuals and depends on the level of physical exertion at the time of exposure to the spores. In a dose response model for Q fever (a bacterial infection caused by *Coxiella burnetii*), Heppell et al. in (38) approximate a distribution for the probability of deposition, *ϕ*, using the Multiple-Path Particle Dosimetry Model (MPPD) software package. Here, we have considered a wide range of values for the deposition probability, between *ϕ* = 10^-4^ and *ϕ* = 0.5. In **Figure 10** we show a wide range of parameter sets involving the value *p*, the deposition probability *ϕ*, the threshold value *M*, and the average rupture size derived from the intracellular model, which all lead to the same value of *r* = 3.31 × 10^-5^. This is the value of *r* obtained by fitting the exponential dose-response curve to the data in **Figure 8**. In this exploration, we are not concerned with exact parameter values, since these will strongly depend on the dose-response data set used to calibrate them. Our aim is to illustrate that, depending on the rupture size distribution used, we can fit the dose-response data with a large number of combinations of the parameters *M*, *ϕ* and *p*. In general, as the threshold value *M* increases, and the average rupture size and deposition probability *ϕ* decrease, the required value of *p* decreases, since more replication will be required to reach the desired threshold *M*.

Our results show the potential to make use of mechanistic intracellular models and dose-response data sets to nest with within-host infection dynamics. If additional experimental data sets were available, a stochastic within-host model could be developed, similar to recent models for *F. tularensis* infection proposed in Refs (16, 25). This approach would not only allow one to compute the probability of a host response to infection, but also the timescale of symptom onset. In order to characterise the timescale of the response, Brookmeyer et al. consider an exponentially distributed delay between the first spore germination and symptom onset, to account for bacterial growth. However, a limitation of this approach is that in considering this delay to have the same distribution for every dose, their model does not incorporate the fact that a higher number of germinating spores would be expected to replicate and produce toxins faster, causing the time to response to be shorter. Wilkening modified the model developed by Brookmeyer et al. to address this issue by including a dose-dependent log-normal distribution to represent the bacterial growth phase (39). Since the data in **Figure 8** only provides the probability of infection from exposure to different doses of spores, we have considered a discrete-time process for the replication and death of extracellular bacteria, where the value of *p* represents how likely bacteria are to die in the early stages of infection (36). However, until more data sets are available, it is not possible to estimate specific within-host parameter values, since different combinations of parameter values can provide the same value of *r* and hence the same dose-response curve. Further experimental work, such as *in vivo* data of the number of spores and bacteria in the lungs and lymph nodes over time, would be needed to calibrate the parameters in a within-host model (or mechanistic dose-response model) of inhalational anthrax infection. Data on anthrax toxin production and stability would also be required in a within-host model of anthrax infection, since the toxins are a key component of pathogenesis. Future work will focus on including the role of toxins in a mathematical model of inhalational anthrax infection.

A restrictive assumption of the intracellular model introduced here is that it only considers a macrophage infection by a single phagocytosed spore. However at higher exposures, it is possible that some macrophages may phagocytose more than one spore. Thus, it would be necessary to include this consideration in the intracellular model. Furthermore, we proposed a linear death rate of intracellular bacteria to keep the model analytically tractable. There is evidence that macrophages with a low intracellular bacterial burden are much more efficient at killing bacteria than those with a higher burden (2, 23, 30). We could generalise our model to include a non-linear, density-dependent death rate of intracellular bacteria, similar to the burden-dependent killing function used in (15). Our model predicts that the intracellular burden of a cell initially infected with a single spore will remain very low, so the inclusion of a density-dependent death rate may be more appropriate when considering higher multiplicities of infection, when each macrophage will phagocytose more than one spore.

To calibrate the parameters of our model, we made use of experimental measurements from a study by Kang et al. (23), of mouse peritoneal macrophage infection with the attenuated non-capsule-producing Sterne strain of *B. anthracis*. This enabled us to mathematically describe a system which can be characterised easily in the laboratory. In fact, one of the more commonly used animal models of anthrax is the AJ mouse model infected with Sterne strain (40). Ideally, for modelling human inhalational anthrax, spores from a fully virulent strain and cells more similar to a human alveolar macrophage would be used. However, this type of data for anthrax is extremely limited. The more clinically relevant alveolar macrophage is complex to isolate and culture, so in the same way that mice are used as a surrogate for primates, peritoneal macrophages are used as a surrogate for the lung’s resident phagocytes. Moreover, the Sterne strain is often used in laboratory settings since it poses a reduced infection risk to laboratory workers, and research with virulent strains of *B. anthracis*, such as the Ames strain, requires enhanced biosafety laboratories (40). However, it can be generally agreed that its value has limitations when modelling disease (40). The capsule is known to protect extracellular bacteria from phagocytosis (14, 41), and thus should be considered when modelling the extracellular dynamics of anthrax infection, which has not been explicitly modelled here. It is possible that the capsule also plays a role in protecting emerging intracellular bacteria from the antimicrobial environment of the host cell, since germinating spores are able to quickly produce and coat themselves in the capsule (42, 43). However, it has been shown that macrophages are still able to kill intracellular bacteria even when they are from a strain that is coated in an antiphagocytic capsule, like the Ames strain (44), and the capsule does not seem to be fully protective against the bactericidal activity of macrophages (45). Therefore, re-parametrising the intracellular model using data from a fully virulent strain would be extremely useful in determining whether the capsule has a significant effect on the intracellular dynamics and fate of a phagocytosed spore.

In conclusion, we have developed and analysed a novel stochastic mathematical model of the intracellular bacterial dynamics of a macrophage infected with a single anthrax spore. By calibrating the model with experimental data, we have found support for a discrete Bernoulli distribution of the spore germination rate, which provides independent evidence for the role of superdormant spores (20, 21). This is both of clinical and biological interest. From a clinical perspective, it indicates the importance to maintain antibiotic dosing for long periods, given the potential for the slow germinating spores to contribute to the characteristic persistence of spores in the lungs after inhalational exposure (46). From a biological perspective, it demonstrates that there might be selective pressure for spores to distribute their germination rates in a heterogeneous manner. This might protect spore populations by ensuring that a reservoir of spores is maintained in case of accidental germination in environments not suitable for growth (47). The results of our calibrated model also predict, in agreement with experimental findings, that many macrophages may be able to recover and resolve the bacterial infection, provided their initial intracellular burden is low. Yet, our results predict a non-zero but low risk of cellular rupture, leading to the release of bacteria from the cell. We believe the intracellular stochastic model proposed here will pave the way to an extension to *in vivo* infection settings and thus, to improve within-host dynamics models.

## Supplemental Data

The **Supplementary Material** contains a detailed explanation of how the data in **Table 1** can be used to find an estimate for the phagocytosis rate of spores. It also contains additional details of the preliminary parameter fitting to the Akoachere et al. data in (26), which allowed us to estimate a prior distribution for the bacteria replication and infected macrophage rupture rates. Finally, the derivation for the likelihood that we have used in the formula for Akaike’s Information Criterion (AIC) has been provided, since it was used to compare the goodness-of-fit between the different germination rate hypotheses considered.

## Data Availability Statement

Computer codes (in Python) for reproducing the results in **Figures 4**–**8**, and **10** are available in Williams et al. (48).

## Author Contributions

All authors conceived the idea and contributed to develop the mathematical models. BW, ML-G, and GL carried out the analysis of the summary statistics in the *Materials and Methods* section. BW and ML-G carried out the Bayesian statistical analysis to carry out parameter calibration with experimental data sets, and designed the results in the *Results* section. BW developed the numerical codes and prepared all the figures. BW and ML-G wrote the first version of the manuscript. All authors contributed to the article and approved the submitted version.

## Funding

BW is supported by an EPSRC CASE studentship, project reference 2345914, in partnership with Dstl under contract number DSTLX-1000142022.

## Conflict of Interest

The authors declare that the research was conducted in the absence of any commercial or financial relationships that could be construed as a potential conflict of interest.

## Publisher’s Note

All claims expressed in this article are solely those of the authors and do not necessarily represent those of their affiliated organizations, or those of the publisher, the editors and the reviewers. Any product that may be evaluated in this article, or claim that may be made by its manufacturer, is not guaranteed or endorsed by the publisher.
