# Supplementary material for: A Stochastic Intracellular Model of Anthrax Infection With Spore Germination Heterogeneity
Source: Front Immunol. 2021 Aug 23;12:688257. doi: 10.3389/fimmu.2021.688257 (PMC8420810; doi:10.3389/fimmu.2021.688257)
Supplement: Supplementary file 1 [file DataSheet_1.pdf]

Supplementary Material: A stochastic intracellular model of anthrax infection with spore germination heterogeneity

Bevelynn Williams, Martín López-García, Joseph J. Gillard, Thomas R. Laws, Grant Lythe, Jonathan Carruthers, Thomas Finnie, Carmen Molina-París

1 ESTIMATION OF THE PHAGOCYTOSIS RATE

We use maximum likelihood estimation (MLE) to find an estimate for the phagocytosis rate of spores, using the data from an *in vitro* study Kang et al. (2005), and reported in Table S1 of this manuscript. As described in the main manuscript, the values in Table S1 are assumed to be the numbers of spores that were phagocytosed within the first 0.5 hours of the experiment, for the three multiplicities of infection (MOI). Since there were the same number of macrophages in the experiment for each MOI, one can consider the same per spore phagocytosis rate, and get a single estimate for this rate. If we assume that each spore is independently phagocytosed at a rate $\rho\ h^{-1}$, then the probability that a given spore has been phagocytosed before time t hours is $1-e^{-\rho t}$. The initial number of extracellular spores is 5×10^5, 10^5, and 5×10^4 for MOI 1:2, 1:10, and 1:20, respectively. Therefore, the likelihood that the intracellular spore counts at 0.5 hours are equal to the values in Table S1, given phagocytosis rate ρ, is

$$L(\rho) = \binom{5\times10^5}{139000}(1-e^{-\frac{\rho}{2}})^{139000}(e^{-\frac{\rho}{2}})^{5\times10^5-139000} \times \binom{10^5}{30500}(1-e^{-\frac{\rho}{2}})^{30500}(e^{-\frac{\rho}{2}})^{10^5-30500} \times \binom{5\times10^4}{13925}(1-e^{-\frac{\rho}{2}})^{13925}(e^{-\frac{\rho}{2}})^{5\times10^4-13925},$$

and the log-likelihood is given by

$$\log L(\rho) = 183425\log(1-e^{-\frac{\rho}{2}}) - 466575\frac{\rho}{2},$$

which is maximised for a value of the per spore phagocytosis rate equal to $\rho = 0.66311\ h^{-1}$. Figure S1 shows the estimated mean number of intracellular spores over the first 0.5 hours of the experiment, with this value of the phagocytosis rate, ρ, for each of the three MOIs.

MOI 1:2	139000
MOI 1:10	30500
MOI 1:20	13925

Table S1. Data taken from Ref. (Pantha et al., 2018, Table 2). Average number of intracellular spores of two replicates of the experiment counted at 1 hour when using spores of a germination deficient strain of anthrax. The number of spores of the germination deficient strain should have remained unchanged between 0.5 hours and 1 hour, because they cannot germinate, and all extracellular spores were removed by washing at 0.5 hours. Thus, after this time, there are no further phagocytosis events.We note that the value for MOI 1:10 reported in Ref. (Pantha et al., 2018, Table 2) was inconsistent with that observed in Ref. (Pantha et al., 2018, Figure 2). We make use of the second one in order to compare our predictions with those by Pantha *et al.*, and this value is more consistent with the trajectory over time for the spore counts in Ref. (Pantha et al., 2018, Figure 2) for MOI 1:10.

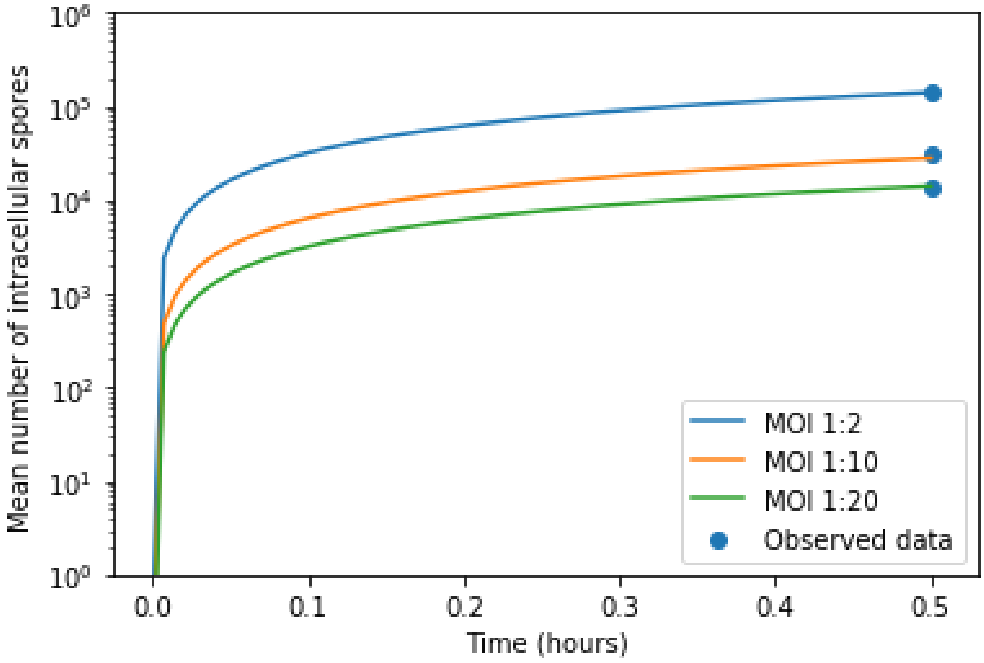

Figure S1. A comparison between model predictions of the mean number of intracellular spores for the first 0.5 hours of the experiments, and the observed number of intracellular spores provided in Table S1. These predictions have been obtained using the estimated per spore phagocytosis rate of $\rho = 0.66311\ h^{-1}$.

2 DETERMINATION OF PRIOR DISTRIBUTIONS WITH DATA FROM AKOACHERE *ET AL.*

We carried out parameter calibration for our model by means of Approximate Bayesian Computation Sequential Monte Carlo (ABC-SMC) (Toni et al. (2009)), and by making use of the spore and bacterial counts measured in experiments (see Kang et al. (2005)). However, some of the model parameters are not identifiable from the measurements of intracellular spore and bacterial counts alone, since, for instance, we are unable to determine whether a reduction in the number of intracellular bacteria is due to bacterial death or macrophage rupture. Therefore, we leveraged rupture time data from Akoachere et al. (2007), in order to inform the selection of some of our prior distributions. Here we explain how this initial choice was made to estimate a potential prior distribution for the replication and rupture rates, λ and γ, respectively.

Akoachere et al. (2007) examined macrophage-spore interactions by fluorescence microscopy. To do this, they used two different dyes, SYTO 13 and PI, which both bind to DNA. SYTO 13 is a green dye that is cell permeable, whereas PI is a red dye that is impermeable to cell membranes. Therefore, when staining macrophages with these dyes, the nucleus of healthy cells shows green fluorescence and the nucleus of dead cells shows red fluorescence. After infecting murine macrophages with *B. anthracis* Sterne strain spores at a spore to macrophage ratio (MOI) of 20:1, they observed 20% PI-positive macrophages at 3.5 hours after exposure, and 90% at 7 hours after exposure, respectively.

In order to compare our model with the observation that 20% of cells had ruptured by 3.5 hours and 90% had ruptured by 7 hours, we need to introduce a delay for phagocytosis in our model, because time $t = 0$ in this experiment represents the time when the macrophages and spores were placed in contact, rather than the time when the spores were phagocytosed. The process with a delay for phagocytosis is shown in Figure S2.

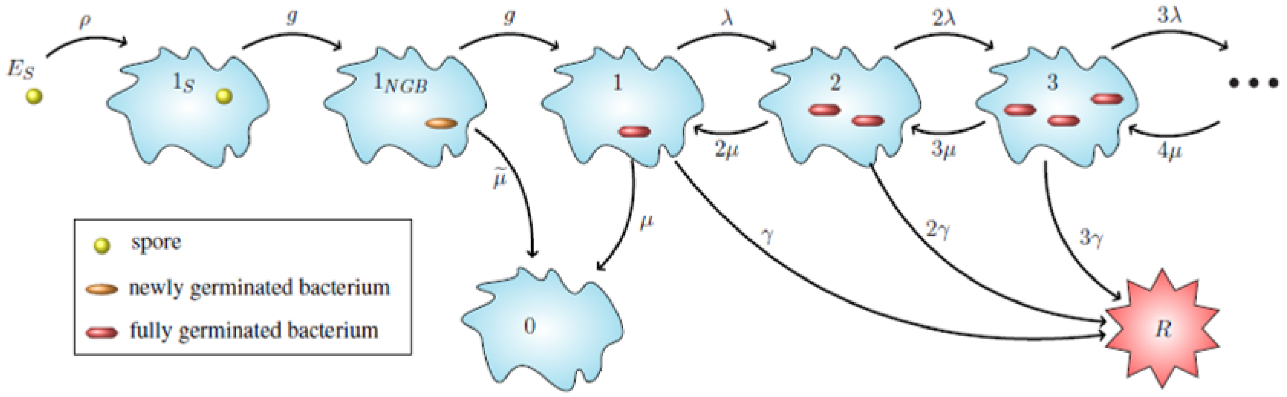

Figure S2. Intracellular infection model with a delay for phagocytosis. State E_S is the initial state, representing one extracellular spore. The per spore phagocytosis rate is $\rho \ hours^{-1}$. State 1_S represents a phagocytosed spore and state 1_{NGB} a newly germinated bacterium (NGB). The germination rate of spore to NGB and the maturation rate of NGB into fully vegetative bacterium for a given spore is denoted by $g \ hours^{-1}$, which leads to an Erlang(2, g) distribution for the germination-maturation time. The rate g is assumed to vary from spore to spore. The death rate of the newly germinated bacterium is given by $\widetilde{\mu} \ hours^{-1}$. States $i \in \mathbb{N} \cup \{0\}$ represent i intracellular bacteria, so that 0 represents recovery, and R represents the rupture of the cell. States 0 and R are absorbing states for the stochastic process. Transitions between states $i \in \mathbb{N}$ represent three types of events: transition to state $i+1$ (division of a bacterium), to state $i-1$ (death of a bacterium), and to state R (rupture of the macrophage with release of i bacteria). The per bacterium division, death, and rupture rates are $\lambda > 0$, $\mu > 0$ and $\gamma > 0$, respectively, all with units $(bacteria \cdot hours)^{-1}$, leading to a linear birth-and-death process with catastrophe. The infected macrophage survives for as long as it does not reach state R.

The process in Figure S2 starting with one extracellular spore has initial condition $X(0) = E_S$. For $i \in \{E_S, 1_S, 1_{NGB}\} \cup \mathbb{N}$, let $p_i(t)$ be the probability that the CTMC is in state i at time t, given that it started in state E_S. That is,

$$p_i(t) = \mathbb{P}(X(t) = i \mid X(0) = E_S).$$

One can write a system of differential equations for these state probabilities, given by

$$\begin{aligned}
\frac{dp_{E_S}}{dt} &= -\rho p_{E_S}, \\
\frac{dp_{1_S}}{dt} &= \rho p_{E_S} - g p_{1_S}, \\
\frac{dp_{1_{NGB}}}{dt} &= g p_{1_S} - (g + \widetilde{\mu}) p_{1_{NGB}}, \\
\frac{dp_1}{dt} &= g p_{1_{NGB}} - (\lambda + \mu + \gamma) p_1 + 2\mu p_2, \\
\frac{dp_i}{dt} &= \lambda(i-1) p_{i-1} + \mu(i+1) p_{i+1} - (\lambda + \mu + \gamma) i p_i, \qquad \text{for } i \geq 2.
\end{aligned}$$

The first three equations can be easily solved to obtain

$$\begin{aligned}
p_{E_S}(t) &= e^{-\rho t},\\
p_{1_S}(t) &= \frac{\rho}{g-\rho}(e^{-\rho t} - e^{-gt}),\\
p_{1_{NGB}}(t) &= \frac{g\rho}{(g-\rho)(g+\widetilde{\mu}-\rho)}e^{-\rho t} - \frac{g\rho}{(g-\rho)\widetilde{\mu}}e^{-gt} + \frac{g\rho}{\widetilde{\mu}(g+\widetilde{\mu}-\rho)}e^{-(g+\widetilde{\mu})t}.
\end{aligned}$$

In order to take into account the heterogeneity in spore germination times previously observed and reported (Setlow (2003, 2013, 2014)), we assume that the germination rate, g, is not a fixed parameter, but varies from spore to spore according to some probability distribution. We consider two different distributions for the germination rate: continuous Gaussian and discrete Bernoulli, as described in the main manuscript.

The time to rupture of a cell following the process in Figure S2 is a random variable, $T^R_{E_S}$. The probability density function for this random variable, given that the spore has germination rate g, is obtained by convolving the function $f_{T^1_{E_S}}(t;g)$, which is the density function for the time to reach state 1, representing a fully vegetative bacteria, with $f_{T^R_1}(t)$, which is the density for the time to reach rupture from state 1. Hence, one has

$$f_{T^R_{E_S}}(t;g) = \int_0^t f_{T^1_{E_S}}(s;g) f_{T^R_1}(t-s)\, ds,$$

where $f_{T^1_{E_S}}(t;g) = g p_{1_{NGB}}(t)$, and $f_{T^R_1}(t)$ is given by Carruthers et al. (2020), as follows

$$f_{T^R_1}(t) = \frac{\gamma(b-a)^2 e^{-\lambda(b-a)t}}{(b-1+(1-a)e^{-\lambda(b-a)t})^2}, \quad t \geq 0, \tag{S1}$$

where $a = \frac{(\lambda+\mu+\gamma)-\sqrt{(\lambda+\mu+\gamma)^2-4\mu\lambda}}{2\lambda}$ and $b = \frac{(\lambda+\mu+\gamma)+\sqrt{(\lambda+\mu+\gamma)^2-4\mu\lambda}}{2\lambda}$. Taking into account the distribution of the germination rate, the overall density function of the rupture time is given by

$$f_{T^R_{E_S}}(t) = \int_0^{+\infty} f_G(g) f_{T^R_{E_S}}(t;g)\, dg,$$

in the case where the germination rate follows a truncated normal distribution, and,

$$f_{T^R_{E_S}}(t) = \varepsilon f_{T^R_{E_S}}(t;g_A) + (1-\varepsilon) f_{T^R_{E_S}}(t;g_B),$$

in the case of the discrete Bernoulli distribution. Then, for both hypotheses, the survival function of a cell starting with one extracellular spore can be written as

$$S^1(t) = \mathbb{P}(X(t) \neq R \mid X(0) = E_S) = 1 - \int_0^t f_{T^R_{E_S}}(t)\, dt.$$

However, since the MOI in the experiment by Akoachere et al. (2007) was a spore to macrophage ratio of 20:1, with many more spores than host cells, our assumption that each cell only phagocytoses one spore might be invalid. Therefore, we consder that at the beginning of the experiment by Akoachere et al. (2007),

each macrophage is surrounded by a "pool" of spores, and that each spore can only be phagocytosed by its nearest cell at the beginning of the experiment. If the suspension of spores and cells is well mixed, then the cell that is closest to each spore will be completely random. We consider that the cells are numbered $i = 1, ..., m$, and the spores are randomly distributed between host cells. Then for each spore, a cell is sampled with replacement so that cell i is chosen with probability $1/m$. Hence, the distribution of spores for all host cells is multinomial, and the number of spores in a given pool (for a fiducial cell) is binomially distributed with parameters $n = 20m$ and $p = 1/m$, since an MOI of 20:1 indicates there are 20 times as many spores as cells. We assume that there are large numbers of spores and cells in the experiment, which means that n will be large and p will be very small. In this limit, a Poisson distribution with mean $np = 20$ provides a good approximation to the binomial distribution for the number of spores in a fiducial pool. Hence, we assume the number of spores in a host cell pool to be Poisson distributed with mean $np = 20$. If we consider one such cell in the experiment, then each of its k surrounding spores will follow the process in Figure S2, and given all rates are linear, the stochastic process for each of the k spores will be independent, up until the time when the first stochastic process enters the rupture state.

Following the argument above, the probability that a cell starting with k surrounding spores has not ruptured by time t is the same as the probability that k processes each starting with one extracellular spore have all not reached the rupture state by time t. That is, $S^k(t) = (S^1(t))^k$, where $S^k(t)$ is the survival function of a cell starting with k extracellular spores in its pool. Thus, we define the probability that a given cell in this experiment has not ruptured before time t by,

$$S(t) = \sum_{k=0}^{+\infty} \frac{20^k e^{-20}}{k!} [S^1(t)]^k.$$

In practise we only include values of k up to $k = 40$ in this sum because $\mathbb{P}(k > 40) < 10^{-4}$.

We used ABC-SMC to calibrate the model parameters with the experimental measurements from Akoachere et al. (2007); namely, 20% of cells had ruptured before 3.5 hours and 90% had ruptured before 7 hours, or equivalently, 80% of cells were still alive at 3.5 hours, and 10% of the cells were alive at 7 hours. We fixed the per spore phagocytosis rate to be the maximum likelihood estimate $\rho = 0.66311\ h^{-1}$. Uniform prior distributions were considered for the remaining parameters, as reported in Table S2. For the model with two types of spores, we fixed $g_A > g_B$ to represent that, without any loss of generality, type A spores are faster to germinate than those of type B. In order to sample parameter values g_A and g_B with prior distributions reported in Table S2, and under the constraint $g_A > g_B$, we follow the ideas from Goggans et al. (2014). We used the Euclidean distance function,

$$[S(3.5) - 0.8]^2 + [S(7) - 0.1]^2,$$

to compare the expected fraction of cells still alive predicted with our analytical expression for the survival function, to the fraction of cells still alive in the experiment at the two time points.

Histograms of the posterior distributions from the final iteration of ABC-SMC are shown in Figure S3 for every model parameter and for both hypotheses. By using the rupture time data, it was possible to learn significantly about the intracellular replication rate λ and the rupture rate γ. Furthermore, these preliminary estimates for both hypotheses lead to a good representation of the rupture dynamics, as shown in Figure S4. Therefore, we used the posterior distributions for these two parameters as prior distributions in our second application of ABC-SMC, with experimental data from Kang et al. (2005) for the time course of the number

Parameter	Units	Description	Prior distribution
Model with continuous germination rate distribution			
μ_g	h^{-1}	Mean of the normal distribution for G	$\log_{10}\mu_g \sim$ U(-2, 1)
σ_g	h^{-1}	Standard deviation of the normal distribution for G	$\log_{10}\sigma_g \sim$ U(-2, 0.15)
$\widetilde{\mu}$	h^{-1}	Death rate of newly germinated bacteria	$\log_{10}\widetilde{\mu} \sim$ U(-4, 1)
λ	$(bacteria \cdot h)^{-1}$	Replication rate of vegetative bacteria	$\log_{10}\lambda \sim$ U(-4,1)
μ	$(bacteria \cdot h)^{-1}$	Death rate of vegetative bacteria	$\log_{10}\mu \sim$ U(-4, 1)
γ	$(bacteria \cdot h)^{-1}$	Rupture rate	$\log_{10}\gamma \sim$ U(-4,1)
Model with discrete Bernoulli germination rate distribution			
ε	-	Probability that a given spore is of type A	$\varepsilon \sim$ U(0, 1)
g_A	h^{-1}	Germination and maturation rate of spores of type A	$\log_{10}g_A \sim$ U(-4, 1)
g_B	h^{-1}	Germination and maturation rate of spores of type B	$\log_{10}g_B \sim$ U(-4, 1)
$\widetilde{\mu}$	h^{-1}	Death rate of newly germinated bacteria	$\log_{10}\widetilde{\mu} \sim$ U(-4, 1)
λ	$(bacteria \cdot h)^{-1}$	Replication rate of vegetative bacteria	$\log_{10}\lambda \sim$ U(-4,1)
μ	$(bacteria \cdot h)^{-1}$	Death rate of vegetative bacteria	$\log_{10}\mu \sim$ U(-4, 1)
γ	$(bacteria \cdot h)^{-1}$	Rupture rate	$\log_{10}\gamma \sim$ U(-4,1)

Table S2. Prior distributions used in the ABC-SMC for the model with continuous heterogeneity of germination rate (top) and the model with two types of spores (bottom).

of intracellular spores and bacteria. This second round of ABC-SMC will allow us to learn about the rest of the model parameters.

The prediction of the function $1 - S(t)$, giving the expected fraction of cells to rupture before time t, is plotted in Figure S4, together with the two data points from Akoachere et al. (2007), for each of the two germination heterogeneity hypotheses. The solid line shows the model prediction using the accepted parameter set that gave the smallest distance in the ABC-SMC, while the shaded region shows the pointwise 95% credible interval of the predictions from the complete posterior distribution.

3 AKAIKE'S INFORMATION CRITERION

To compare the goodness-of-fit between the two different germination rate hypotheses considered, we used Akaike's information criterion (AIC), which penalises models with a higher number of parameters if there is not enough improvement in the goodness-of-fit to warrant the additional complexity. Since we only had 32 data points in total to compare the model predictions to, we used a form of the AIC that is corrected for small sample sizes. The formula for this is given by Burnham and Anderson (2002), as follows

$$\text{AIC}_C = -2\log(\mathcal{L}(\underline{\hat{\theta}}, \hat{\sigma})) + \frac{2Kn}{n-K-1}.$$

In this formula, $\log(\mathcal{L}(\underline{\hat{\theta}}, \hat{\sigma}))$ is the maximised log-likelihood, n is the number of data points, and $K = r+1$, where r is the dimensionality of our model parameter space. We assume that once one log-transforms the observed data and the predicted values from our model, the errors are normally distributed. We let

$$\log(d(t)) = \log(m(\underline{\theta}, t)) + \varepsilon_t \qquad \text{for } t \in T,$$

where $d(t)$ is the experimental data point at time t, and $m(\underline{\theta}, t)$ is the predicted model output at the same time, for each of the n time points in $T = \{t_1, t_2, \ldots, t_n\}$. The errors, ε_t, are assumed to be independent, normally distributed with a constant variance σ^2. Therefore, these residuals have the following joint

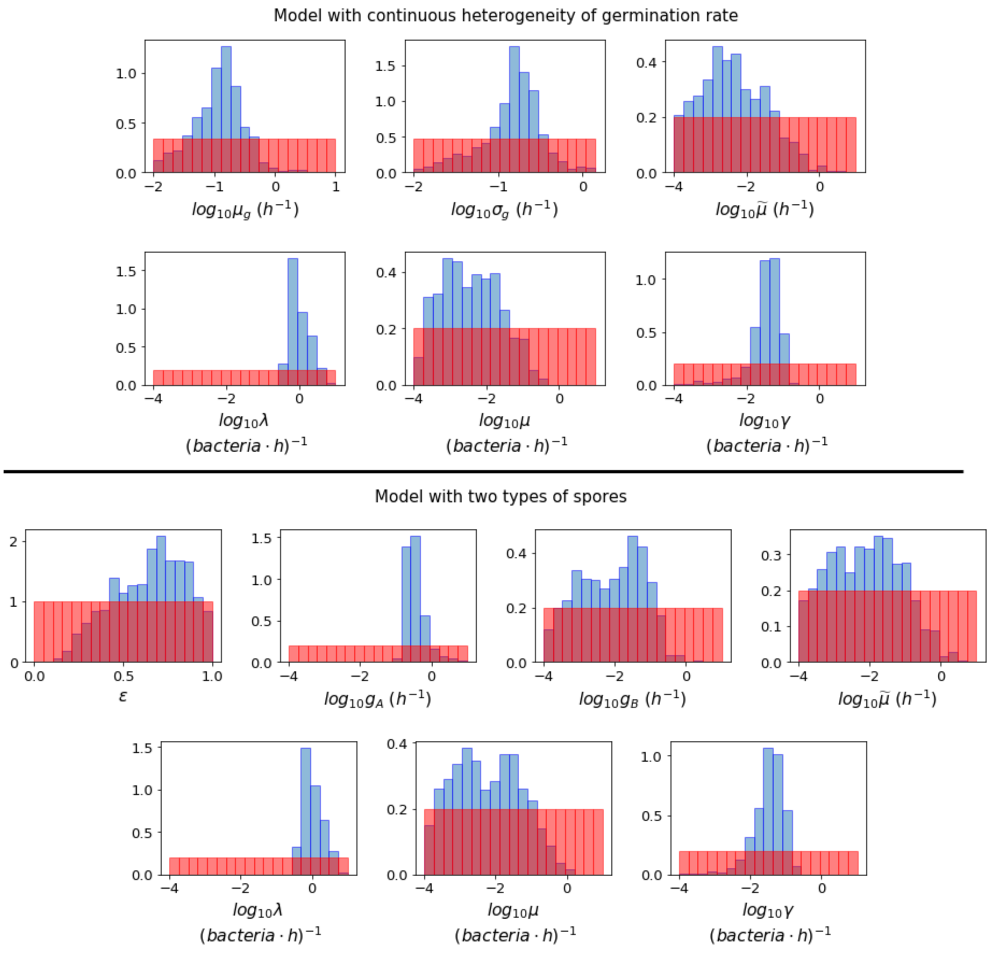

Figure S3. Prior distributions considered (red) and posterior histograms obtained (blue) when performing ABC-SMC with data from Akoachere et al. (2007) of the proportion of dead macrophages at two different time points, for the model with continuous heterogeneity in the germination rate (top), and the model with two types of spores (bottom).

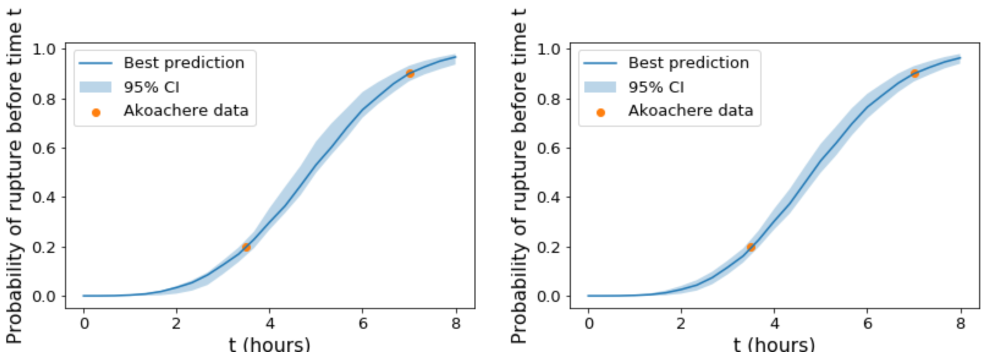

Figure S4. Best predictions (solid lines) and pointwise 95% credible intervals (shaded regions) of the fraction of cells that would be expected to rupture before time t in an experiment with MOI 20:1, compared to data from Akoachere et al. (2007), for the model with two types of spores (left), and the model with continuous distribution for the germination rate (right). The best prediction is the model output obtained by using the accepted parameter set with the smallest distance from the data. The pointwise 95% credible intervals show the uncertainty in the predictions given the posterior distributions for the model parameters.

probability distribution

$$f(\underline{\varepsilon} \mid \underline{\theta}, \sigma) = \prod_{t \in T} \frac{1}{\sqrt{2\pi\sigma^2}} e^{-\frac{1}{2}(\frac{\varepsilon_t}{\sigma})^2},$$

and hence the likelihood is,

$$\mathcal{L}(\underline{\theta}, \sigma) = \left(\frac{1}{\sqrt{2\pi\sigma^2}}\right)^n e^{-\frac{1}{2}\sum_{t \in T}(\frac{\varepsilon_t}{\sigma})^2}.$$

Then the log-likelihood is given by

$$\log(\mathcal{L}(\underline{\theta}, \sigma)) = -n\log(\sqrt{2\pi}) - n\log(\sigma) - \frac{1}{2\sigma^2}\sum_{t \in T} \varepsilon_t^2.$$

The Approximate Bayesian Computation Sequential Monte Carlo (ABC-SMC) algorithm was used for the parameter calibration to obtain parameter estimates, $\hat{\underline{\theta}}$, which minimise $\sum \hat{\varepsilon}_t^2$, and hence maximise the log-likelihood with respect to the errors ε_t. To maximise the log-likelihood with respect to σ, one can differentiate with respect to σ and equate to 0, giving the MLE of σ^2 to be

$$\hat{\sigma}^2 = \frac{\sum_{t \in T} \varepsilon_t^2}{n}.$$

Thus, the maximised log-likelihood is

$$\log\big(\mathcal{L}(\hat{\underline{\theta}}, \hat{\sigma})\big) = -\frac{1}{2} n\log(\hat{\sigma}^2) - \frac{n}{2}\log(2\pi) - \frac{n}{2}.$$

The additive constants can be discarded because they will be equal for all models with the same data set. Thus, we obtain

$$\log\big(\mathcal{L}(\hat{\underline{\theta}}, \hat{\sigma})\big) = -\frac{1}{2} n\log(\hat{\sigma}^2).$$

Then the final formula that we used for the AIC is

$$\text{AIC}_C = n\log\left(\frac{\text{SSR}}{n}\right) + \frac{2Kn}{n - K - 1}, \tag{S2}$$

where n is the number of data points and K the number of parameters to be estimatated, including the r model parameters and the variance of the residuals, σ^2. In this formula, SSR is the sum of the squared residuals, given by,

$$\text{SSR} = \sum_{t \in T} \big[\log(d(t)) - \log(m(\hat{\underline{\theta}}, t))\big]^2.$$

REFERENCES

Kang TJ, Fenton MJ, Weiner MA, Hibbs S, Basu S, Baillie L, et al. Murine macrophages kill the vegetative form of Bacillus anthracis. *Infection and immunity* **73** (2005) 7495–7501.

Pantha B, Cross A, Lenhart S, Day J. Modeling the macrophage-anthrax spore interaction: Implications for early host-pathogen interactions. *Mathematical biosciences* **305** (2018) 18–28.

Toni T, Welch D, Strelkowa N, Ipsen A, Stumpf MP. Approximate Bayesian computation scheme for parameter inference and model selection in dynamical systems. *Journal of the Royal Society Interface* **6** (2009) 187–202.

Akoachere M, Squires RC, Nour AM, Angelov L, Brojatsch J, Abel-Santos E. Identification of an in vivo inhibitor of Bacillus anthracis spore germination. *Journal of Biological Chemistry* **282** (2007) 12112–12118.

Setlow P. Spore germination. *Current opinion in microbiology* **6** (2003) 550–556.

Setlow P. Summer meeting 2013–when the sleepers wake: the germination of spores of Bacillus species. *Journal of applied microbiology* **115** (2013) 1251–1268.

Setlow P. Germination of spores of Bacillus species: what we know and do not know. *Journal of bacteriology* **196** (2014) 1297–1305.

Carruthers J, Lythe G, López-García M, Gillard J, Laws TR, Lukaszewski R, et al. Stochastic dynamics of Francisella tularensis infection and replication. *PLOS Computational Biology* **16** (2020) e1007752.

Goggans PM, Cao L, Henderson RW. Assigning priors for parameters constrained to a simplex region. *AIP Conference Proceedings* (American Institute of Physics) (2014), vol. 1636, 94–99.

Burnham K, Anderson D. Model selection and multimodel inference: a practical information-theoretic approach, second edition. *New York, USA: Springer* (2002).
